# Deciphering the Methylation Landscape in Breast Cancer: Diagnostic and Prognostic Biosignatures through Automated Machine Learning

**DOI:** 10.3390/cancers13071677

**Published:** 2021-04-02

**Authors:** Maria Panagopoulou, Makrina Karaglani, Vangelis G. Manolopoulos, Ioannis Iliopoulos, Ioannis Tsamardinos, Ekaterini Chatzaki

**Affiliations:** 1 Laboratory of Pharmacology, Medical School, Democritus University of Thrace, GR-68100 Alexandroupolis, Greece; mpanagop@med.duth.gr (M.P.); mkaragla@med.duth.gr (M.K.); emanolop@med.duth.gr (V.G.M.); 2 Department of Basic Sciences, School of Medicine, University of Crete, GR-71003 Heraklion, Greece; iliopj@med.uoc.gr; 3 JADBio, Gnosis Data Analysis PC, Science and Technology Park of Crete, GR-70013 Heraklion, Greece; tsamard@csd.uoc.gr; 4 Department of Computer Science, University of Crete, GR-70013 Heraklion, Greece; 5 Institute of Applied and Computational Mathematics, Foundation for Research and Technology–Hellas, GR-70013 Heraklion, Greece; 6 Institute of Agri-Food and Life Sciences, Hellenic Mediterranean University Research Centre, GR-71410 Heraklion, Greece

**Keywords:** breast cancer, methylation, machine learning, signature, predictive model, bioinformatics, pathway, transcription

## Abstract

**Simple Summary:**

Breast cancer (BrCa) is characterized by aberrant DNA methylation. We leveraged high-throughput methylation data from BrCa and normal breast tissues and identified 11,176 to 27,786 differentially methylated genes (DMGs) against clinically relevant end-points. Innovative automated machine learning was employed to construct three highly performing signatures for (1) the discrimination of BrCa patients from healthy individuals, (2) the identification of BrCa metastatic disease and (3) the early diagnosis of BrCa. Furthermore, functional analysis revealed that most genes selected in the signatures showed associations to BrCa, with regulation of transcription being the main biological process, the nucleus being the main cellular component and transcription factor activity and sequence-specific DNA binding being the main molecular functions. Overall, revisiting methylome datasets led to three high-performance signatures that are readily available for improving BrCa precision management and significant knowledge mining related to disease pathophysiology.

**Abstract:**

DNA methylation plays an important role in breast cancer (BrCa) pathogenesis and could contribute to driving its personalized management. We performed a complete bioinformatic analysis in BrCa whole methylome datasets, analyzed using the Illumina methylation 450 bead-chip array. Differential methylation analysis vs. clinical end-points resulted in 11,176 to 27,786 differentially methylated genes (DMGs). Innovative automated machine learning (AutoML) was employed to construct signatures with translational value. Three highly performing and low-feature-number signatures were built: (1) A 5-gene signature discriminating BrCa patients from healthy individuals (area under the curve (AUC): 0.994 (0.982–1.000)). (2) A 3-gene signature identifying BrCa metastatic disease (AUC: 0.986 (0.921–1.000)). (3) Six equivalent 5-gene signatures diagnosing early disease (AUC: 0.973 (0.920–1.000)). Validation in independent patient groups verified performance. Bioinformatic tools for functional analysis and protein interaction prediction were also employed. All protein encoding features included in the signatures were associated with BrCa-related pathways. Functional analysis of DMGs highlighted the regulation of transcription as the main biological process, the nucleus as the main cellular component and transcription factor activity and sequence-specific DNA binding as the main molecular functions. Overall, three high-performance diagnostic/prognostic signatures were built and are readily available for improving BrCa precision management upon prospective clinical validation. Revisiting archived methylomes through novel bioinformatic approaches revealed significant clarifying knowledge for the contribution of gene methylation events in breast carcinogenesis.

## 1. Introduction

Breast cancer (BrCa) is the most common cancer among women, with over 2 million new cases in 2018 [1]. BrCa is characterized by high survival rates, which decrease upon distant metastasis formation [2]. It is well established that not only genetic, but also epigenetic changes occur during BrCa initiation and progression. DNA methylation is the most studied epigenetic mechanism that is highly involved in the regulation of gene expression in BrCa cells [3]. Aberrant methylation in BrCa tissues or in liquid biopsies has been characterized and correlated to diagnosis [4,5,6], prognosis [7,8,9] and the prediction of treatment response [10,11], leading to emerging biomarkers in the BrCa management cascade. Recently, the introduction of genome-wide methylation analyses enables the simultaneous study of up to hundreds of thousands of CpG sites, and produces high-dimensional datasets which can allow in-depth understanding of the events of methylation in BrCa pathogenesis, as well as its translation to clinical solutions.

As big “-omics” data have accumulated, methods for their exploitation have evolved rapidly. Machine learning (ML), using a variety of algorithms which perform intelligent predictions, has led to increasingly penetrating biomarker discoveries in cancer [12,13]. Automated tools for ML (AutoML) have recently become available, which promise to democratize data analysis by making it available to non-experts, drastically increase productivity, improve the replicability of statistical analysis, facilitate the interpretation of results, and shield against common methodological analysis pit-falls such as overfitting [14]. Along with other bioinformatic tools performing functional analysis, researchers in the scientific field of translational medicine and molecular biology are able to extract the maximum information from laborious and expensive array examinations of precious and scarce clinical samples, leading to personalized clinical decisions and disease management.

**Figure 1:**
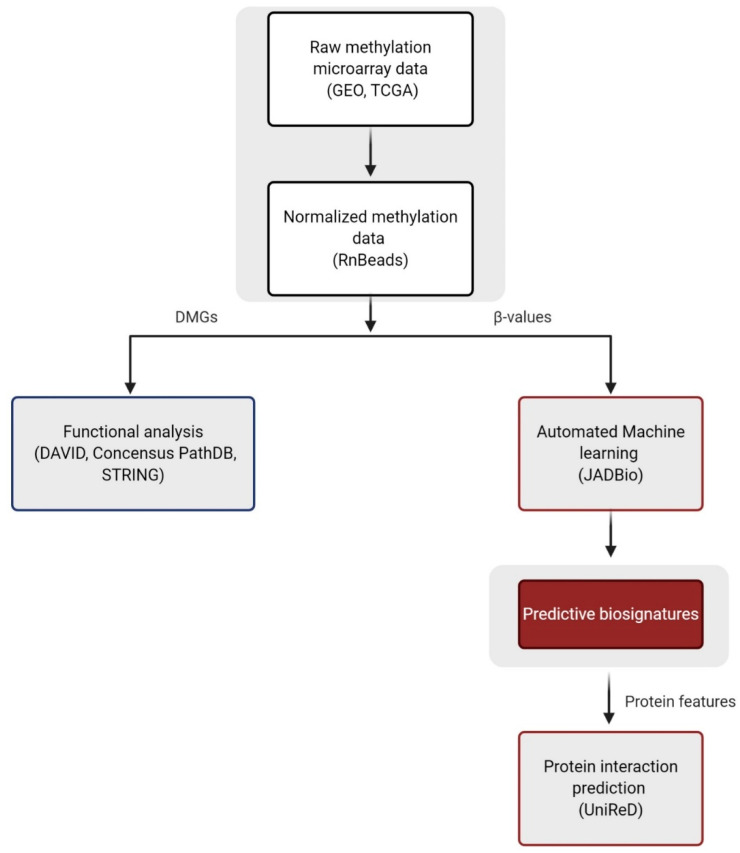
Study workflow. Abbreviations: DMGs = differentially methylated genes, GEO = Gene Expression Omnibus, TCGA = The Cancer Genome Atlas. Created with BioRender.com, accessed on 8 February 20.

ML has been employed for improving BrCa diagnosis [15,16] and prognosis [9,17] in different types of datasets; however, to the best of our knowledge, only List et al. [18] has applied ML to BrCa methylation data, although still failing to construct an accurate model to classify disease subtypes. In our opinion, there is still much to gain by exploiting ML approaches in analyzing genome-wide methylation BrCa datasets, both for knowledge mining as well as to construct clinically relevant models. To this end, in the present study we used publicly available high-throughput methylation datasets from readings by the Illumina methylation 450 bead-chip array, found in The Cancer Genome Atlas (TCGA) program and the Gene Expression Omnibus (GEO) databases and performed a multi-task bioinformatic analysis (Figure 1). Retrieved BrCa and normal tissue methylation data were allocated into groups according to five major clinical endpoints related to prognosis and diagnosis. Differentially methylated genes (DMGs) identified by differential methylation analysis were subjected to further functional analysis in order to identify epigenetically regulated pathways and functions in the pathophysiology of BrCa. Most importantly, ad hoc AutoML technology, specially designed for analyzing high-dimensional low-number -omics datasets [14], was applied in order to build accurate diagnostic and prognostic signatures, adding clinical value in the personalized management of BrCa. Our AutoML results were additionally validated through a different approach, using a text mining tool for the prediction of protein interactions [19].

## 2. Results

### 2.1. Differential Methylation between BrCa and Normal Breast Tissue

**Figure 2:**
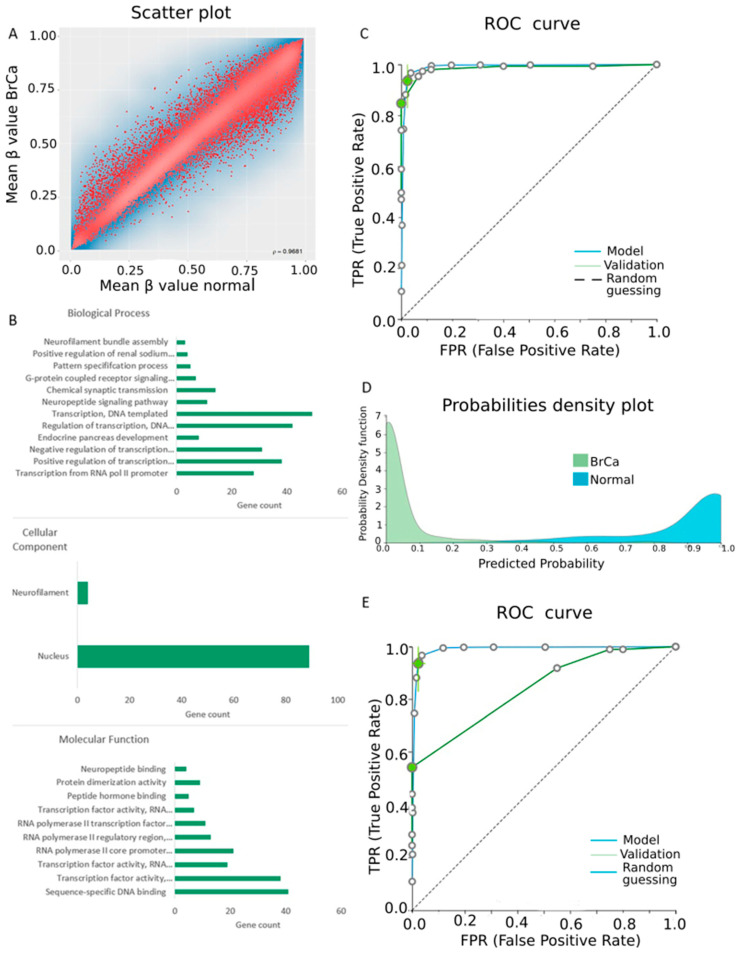
Differential methylation between breast cancer (BrCa) and normal breast tissue. (**A**) A scatterplot showing the mean β value for each gene among BrCa and normal tissues. (**B**) The gene ontology analysis of top 250 differentially methylated genes (DMGs) in the aspects of molecular function, biological process and cellular component analysis. (**C**) Receiver operating characteristic (ROC) curves of training (blue line) and validation (green line) models. (**D**) Probability density plot depicts discrete distributions among studied classes of the training group. (**E**) ROC curves of training (blue line) and external validation (green line) models. Abbreviations: BrCa = breast cancer, DMGs = differentially methylated genes, ROC = receiver operating characteristic.

In the first analysis, raw methylome data from 520 BrCa (primary and metastatic) and 185 normal breast tissues were analyzed by means of RnBeads and 27,786 DMGs (false discovery rate (FDR) < 0.05) were revealed. Figure 2A depicts a scatterplot showing mean β values for each gene analyzed. Array gene methylation between the two groups was overall closely correlated (ρ = 0.9681). DMGs between the two groups were automatically ranked by RnBeads and the 250 top-ranking genes were chosen for further functional analysis. Of these DMGs, only 10 were hypomethylated and the remaining 240 DMGs were hypermethylated in BrCa in relation to normal tissues. The complete list of the 250 top-ranking differentially methylated genes between BrCa and normal tissues is presented in Table S1.

Gene ontology analysis of the 250 DMGs was carried out using the DAVID tool (Figure 2B). Molecular function analysis showed enrichment in sequence-specific DNA binding, in transcription factor activity and in the RNA polymerase II core promoter proximal region. For biological process enrichment analysis, DMGs were found to participate mainly in transcription from RNA pol II promoter, endocrine pancreas development, regulation of transcription and DNA templating. Finally, cellular component analysis showed mainly a nucleus enrichment of the studied genes. Table S2 contains enrichment percentages and gene counts derived frin DAVID analysis. KEGG and Reactome analysis via ConsensusPathDB mainly revealed enrichment in signal transduction and metabolism (Figure S1A). The protein–protein interaction (PPI) network of the 250 DMGs was visualized using the Cytoscape tool and is demonstrated in Supplementary File S1.

β-values produced by RnBeads were analyzed by JADBio in order to construct diagnostic models. The original dataset (520 primary and metastatic BrCa and 185 normal tissues) was automatically and randomly split into a training dataset of 367 BrCa and 125 normal tissues and a validation dataset of 151 BrCa and 60 normal tissues. Analysis of the training dataset of 28,581 gene array features produced one signature containing five features via a classification random forests algorithm. Four of them were long non-coding RNA (lncRNA) genes, namely *AC104435.5, AC002550.1, AC124283.3* and *AC078802.1,* and the last was the pseudogene *DND1P1* (Table 1). In discriminating health from disease, this signature reached an area under the curve (AUC) of 0.994 (0.982–1.000) (Figure 2C). Upon validation, the model showed an AUC of 0.988 (Figure 2C), verifying the stability and accuracy of the model’s performance. The model’s performance and inspection results are depicted in Figure 2D and Figure S1B–D. To further verify the level of discrimination of our 5-feature signature, we applied it to an external, unrelated dataset. Upon external validation, the model showed an AUC of 0.888 (Figure 2E), verifying the predicted performance in discriminating health from BrCa.

### 2.2. Differential Methylation between Primary and Metastatic BrCa

**Figure 3:**
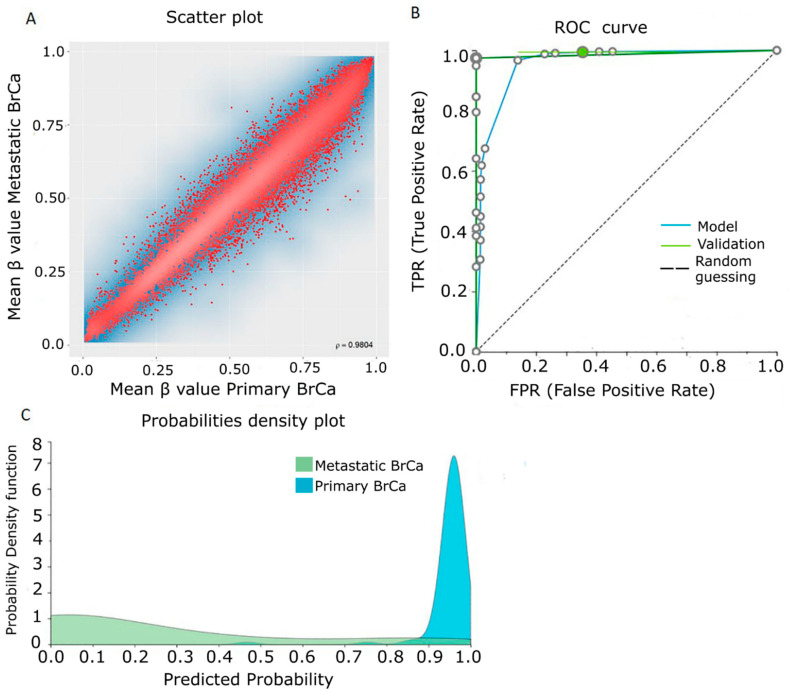
Differential methylation between primary and metastatic BrCa. (**A**) Scatterplot showing mean β value for each gene among primary BrCa and metastatic BrCa. (**B**) ROC curves of training (blue line) and validation (green line) models. (**C**) Probability density plot depicting distinct distributions among studied classes of training group. Abbreviations: BrCa = breast cancer, DMGs = differentially methylated genes, ROC = receiver operating characteristic.

Methylomes of primary BrCa were analyzed in comparison to those from metastatic BrCa in order to detect changes related to metastatic transformation. Raw data from 132 primary cancers and 31 cancers with distant metastasis were analyzed using RnBeads and 24,638 DMGs (FDR < 0.05) were detected. Figure 3A depicts a scatterplot showing the mean β values for each gene analyzed, showing that methylation levels between the two groups were highly correlated (ρ = 0.9804).

Then, the 250 top-ranking DMGs were further subjected to enrichment analysis. A total of 126 of them were hypermethylated and 124 were hypomethylated in metastatic BrCa in relation to primary BrCa. The complete list of the 250 top-ranking differentially methylated genes between primary and metastatic BrCa tissues is presented in Table S3. Gene ontology analysis by DAVID did not show any statistically significant correlation in terms of biological process, molecular function and cellular component. Similarly, KEGG and Reactome pathway analysis by ConsensusPathDB did not lead to a pathway. The PPI network of the 250 DMGs is demonstrated in Supplementary File S2.

β-values of each gene were analyzed by JADBio in order to construct a diagnostic/prognostic model. The original dataset was automatically and randomly split into a training dataset of 93 primary and 21 metastatic tissues and a validation dataset of 39 primary and 10 metastatic tissues. AutoML analysis of the 28,730-feature training dataset produced, via a support vector machines algorithm, one signature containing three features, including two lncRNA genes *(AL139011.1* and *AD000671.3*) and the protein coding gene *USP16* (Table 1). In the training dataset, this signature exhibited an AUC of 0.986 (0.921–1.000) in discriminating primary from metastatic disease (Figure 3B) and in the validation dataset the AUC was 0.992 (Figure 3B), verifying the accurate estimation of the model’s projected performance (Figure 3C and Figure S2A–C).

### 2.3. Differential Methylation between Stage I BrCa and Normal Breast Tissue

**Figure 4:**
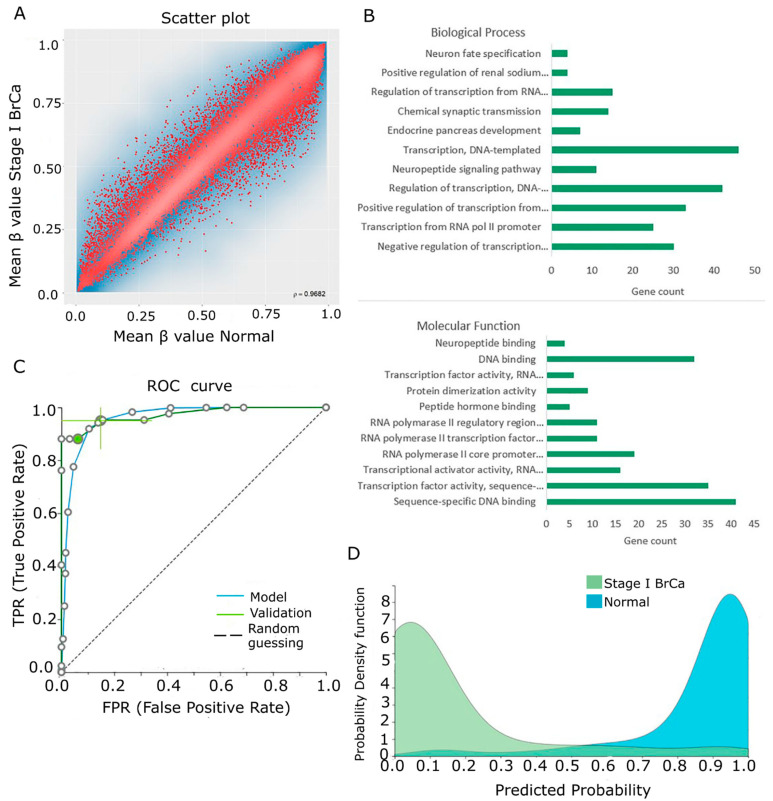
Differential methylation between early-stage BrCa and normal breast tissue. (**A**) Scatterplot showing mean β values for each gene among Stage-I BrCa and normal tissues. (**B**) The gene ontology analysis of the top 250 DMGs in the areas of molecular function, biological process and cellular component analysis. (**C**) ROC curves of training (blue line) and validation (green line) models. (**D**) Probability density plot depicting separated distributions among studied classes of the training group. Abbreviations: BrCa = breast cancer, DMGs = differentially methylated genes, ROC = receiver operating characteristic.

In order to detect early methylation events in the BrCa carcinogenetic process, methylome raw data from 136 stage-I BrCa and 111 normal breast tissues were subjected to RnBeads differential methylation analysis. A total of 26,046 DMGs (FDR < 0.05) were detected. A scatterplot (ρ = 0.9682) showing mean β values for each gene in Stage-I BrCa versus normal tissues is depicted in Figure 4A. Next, the 250 top-ranking genes (13 hypomethylated and 237 hypermethylated in Stage-I cancer in relation to normal) were chosen for further functional analysis. The complete list of the 250 top-ranking differentially methylated genes between BrCa Stage-I and normal tissues is presented in Table S4. Biological process analysis showed enrichment in transcription, DNA-templated synthesis, regulation of transcription and positive or negative regulation of transcription from RNA pol II promoter. The following molecular functions were also enriched: sequence-specific DNA binding, transcription factor activity and DNA binding (Figure 4B). Finally, cellular component analysis showed only nucleus enrichment. Table S5 contains the enrichment percentages and gene counts from DAVID analysis. KEGG and Reactome analysis showed that DMGs were mainly enriched in signal transduction, metabolism and signaling by GPCR (Figure S3A). The PPI network of the 250 DMGs is presented in Supplementary File S3.

Then, β-values were uploaded to JADBio in order to construct early diagnostic models. The original dataset was randomly split into a training dataset of 94 Stage-I BrCa and 79 normal tissues and a validation dataset of 42 Stage-I BrCa and 32 normal tissues. From the 28,702-feature training dataset, AutoML produced six equivalent signatures of five features each via the support vector machine algorithm. Signatures showed an AUC of 0.973 (0.920–1.000) in discriminating early disease (Figure 4C). Common features among them included one protein coding gene (*AIM2*), two lncRNA genes (*AL513008.1* and *AC004884.2*) and one long intergenic non-protein coding RNA (lincRNA) gene (*LINC01563)*. The non-common features among them were five protein coding genes (*DNM2, SSH1, PDGFRB, TIMP3* and *AP2M1*) and one lincRNA gene (*LINC00623*) (Table 1). Upon validation, this performance reached a range in the AUC of 0.972–0.984 (Figure 4C), verifying the stability and accuracy of its estimation. Model and performance validation and inspection are depicted in Figure 4D and Figure S3B–D.

### 2.4. Differential Methylation between Early- and Advanced-Stage BrCa

**Figure 5:**
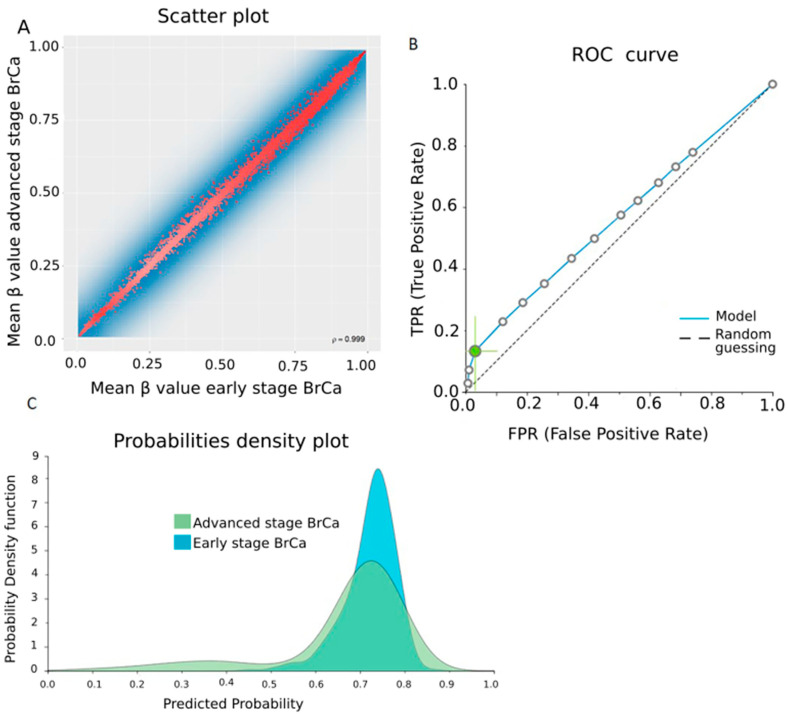
Differential methylation between early- and advanced-stage BrCa. (**A**) A scatterplot showing mean β values for each gene in the early and advanced BrCa stages. (**B**) ROC curve of the training model. (**C**) Probability density plot depicting overlapping distributions among studied classes in the training group. Abbreviations: BrCa = breast cancer, DMGs = differentially methylated genes, ROC = receiver operating characteristic.

Next, in order to detect important methylation events related to the progression of BrCa to advanced-stage disease, we conducted an analysis of raw methylome data from 521 early and 221 advanced BrCa patients, identifying 11,176 DMGs (FDR < 0.05). Figure 5A depicts a scatterplot showing the mean β values for each gene and that methylation between the two groups was overall closely correlated (ρ = 0.999). Based on the 250 top-ranking genes, 119 were hypomethylated and the remaining 131 DMGs were hypermethylated in advanced disease in relation to early disease. Functional analysis by DAVID did not show any statistically significant correlation as far as biological process, molecular function and cellular component analysis concerns. Similarly, KEGG and Reactome pathway analysis did not lead to any pathways. The PPI network of 250 DMGs is depicted in Supplementary File S4.

To deliver predicting signatures, the original dataset was randomly split into a training dataset of 366 early and 152 advanced BrCa samples and a validation dataset of 155 early and 69 advanced BrCa samples. AutoML analysis of the 28,637-feature training dataset produced a five-feature signature via the support vector machine algorithm. Features included four protein coding genes, namely *SMARCAD1, RWDD4, RPF2* and *WDR11* and one lncRNA gene, *SNHG25* (Table 1). The signature’s performance in discriminating early- from advanced-stage disease was poor, showing an AUC of 0.559 (0.513–0.600) (Figure 5B,C and Figure S4A,B). Increasing the cutoff number of features in the signature to 25 did not result in a better performing signature, with an AUC of 0.575 (0.526–0.622).

### 2.5. Survival Analysis of Primary BrCa Patients

Finally, in order to build a methylation-based prognostic signature, AutoML time to event (survival) analysis was performed using raw methylome data from 894 patients—626 and 268 primary BrCa patients were randomly allocated into the training and validation datasets, respectively. AutoML analysis in the 28,635-feature training dataset led to four equivalent prognostic signatures of five features each, via the ridge Cox regression algorithm. The concordance index was 0.592 (0.544–0.641), demonstrating poor prognostic performance. The four common genes in the signatures were two lncRNAs (*AP005436.3* and *DDN-AS1*), an lincRNA (*XX-C2158C12.2*) and a protein coding gene (*IL17RE*). The non-common genes were an lncRNA (*AL355916.2*), an lincRNA (*LINC00824*) and two protein coding genes (*NET1* and *BRINP2*) (Table 1). Once again, increasing the cutoff number of features in the signature to 25 resulted in a slightly increased but still poor prognostic power, with an AUC of 0.606 (0.558–0.650).

### 2.6. Biological Associations of Identified Proteins with BrCa

In order to examine if the identified proteins included in the signatures were somehow implicated in BrCa biology, we crosschecked our results using another bioinformatic tool for protein interaction prediction, called UniReD. UniReD is a text mining tool that predicts functional associations of proteins. Two proteins, RWDD4 and BRINP2, were excluded from the analysis as UniReD could not provide any information on them. All the other protein features included in the signatures were found to be associated to breast cancer pathways (according to the KEGG pathway identification). Furthermore, using a list of 10 genes that are known to be related to breast cancer biology—*BRCA1*, *BRCA2*, *RASSF1*, *ESR1*, *TP53*, *PIK3CA*, *BRMS1*, *CDH1*, *CST6, PTEN* (Table S6)—we examined whether UniReD could find any functional association with the proteins included in the signatures (Table 1). Notably, all of the proteins showed some associations to these BrCa genes and were ranked accordingly (Table 2). TIMP3, PDGFRB and DNM2, all included in the biosignature specific for early-stage disease, showed the closest association, with TIMP3 found to be related to all BrCa genes examined.

## 3. Discussion

DNA methylation is a key regulator of gene expression in mammalian cells. The disruption of DNA methylation machineries contributes to cancer biology, leading to abnormal expression of tumor-related genes involved in metastasis, immune escape and metabolism [20,21]. However, the exact methylation events and their critical timing during carcinogenesis and tumor progression are not fully described. Nevertheless, the clinical exploitation of aberrant methylation patterns in malignant breast tissue or in liquid biopsy material is attracting increasing interest for biomarker research and development projects. Recently, as whole-genome methylation arrays have become available, several studies have been conducted in breast cancer [22,23,24]. Their valuable methylome readings are archived and are accessible for further in-depth analysis for knowledge mining.

Revisiting a given experimental observation is scientifically essential for maximum conclusion extraction as new and powerful statistical and computational methods are introduced. Numerous bioinformatic studies analyzing high-dimensional datasets of various modalities [25,26,27] have produced significant knowledge for BrCa biology, whereas applications of ML approaches have recently become spearheads for building powerful classifiers with major advantages towards diagnostic clinical applications [9,28,29]. Here, our ambition has been to exploit genome-wide BrCa methylation datasets through bioinformatic analysis using readily available tools in order to identify DMGs, to reveal pathophysiological implications by functional analysis and most importantly to build accurate and simple predictive signatures by means of feature selection, to be exploited in personalized BrCa management.

The primary contribution of this effort is the delivery of three accurate and low-feature-number signatures for BrCa diagnosis and prognosis, through the application of an innovative validated AutoML technology in high-dimensional methylome datasets. We employed JADBio, an ad-hoc platform for biomedical studies, designed to deliver high-quality predictive and diagnostic models, employing standard, best-practice and state-of-the-art statistical and machine learning methods. JADBio identifies multiple (in the case of biological redundancy) equivalent biosignatures upon feature selection and provides accurate, non-optimistic estimates of maximum predictive performance [14]. JADBio has previously been successfully used to produce signatures for clinical applications such as the development of lung cancer between smokers [29] or suicide amongst depressive patients [30]. Only recently, by revisiting publicly available -omics datasets via JADBio, we were able to deliver accurate highly-performing blood-based predictive biosignatures in Alzheimer’s disease [28] and classifiers for metastatic BrCa based on novel circulating cell free DNA methylation patterns [9].

Using this AutoML tool, we were able to construct three well-performing accurate biosignatures from available methylomes, via support vector machines and random forest classification algorithms, presenting two advantages of major significance for further developments in biomarker discovery: (1) A low feature number via feature selection, i.e., automatic calculations for identifying the minimum feature number within a dataset of some thousands of features that retain the maximum classifying power. Reducing the dimensions of a signature is a great advantage in terms of translatability to cost-effective assays with less of a need for multiplexing, moving from the multi-dimensional -omics results to simpler classifiers. Upon prospective clinical validation, these signatures can offer feasible solutions for laboratory tests that could be realized in any standard equipped diagnostic lab. (2) Stable performance of the models upon validation, adding credibility if they are selected for clinical development. JADBio has been shown to shield against typical methodological pitfalls in data analysis that lead to overfitting and overestimating performance and therefore to misleading results [14,31]. This is again confirmed here, as the AUC of the biosignatures built did not fall significantly when tested in the validation subdatasets or in independent cohorts.

In particular, in our analysis of BrCa methylomes vs. normal tissues, a five-gene signature emerged, exhibiting a high AUC of 0.994 (0.982–1.000), which was verified upon validation. Four of the genes included were lncRNAs. LncRNAs are non-coding RNAs, containing more than 200 nucleotides. Studies have shown that lncRNAs play an important role in many biological processes, such as epigenetic mechanisms, gene transcription, cell cycle and cell differentiation [32]. Their role has also been reported in cancer initiation and progression, but limited knowledge on these is available to date [33]. Our findings that lncRNAs can accurately discriminate BrCa from health indirectly support their contribution to tumor biology. The lncRNA AC078802.1 is an antisense to *ACTRT3* and its aberrant expression has only been demonstrated in squamous lung cancer, presenting contradictory results so far [34,35]. Our methylome bioinformatics analysis revealed that AC078802.1 is hypermethylated in BrCa, opening the field for further analysis. To our knowledge, the remaining lncRNAs identified in this signature, as well as the pseudogene *DND1P1*, have never been reported in cancer research and following this report await further investigation.

In order to construct a model specific for the identification of metastatic BrCa disease, a three-gene signature was built, with a significant AUC of 0.986 (0.921–1.000). Two lncRNAs included in the signature were found to be hypermethylated in metastatic BrCa as compared to primary BrCa. The third feature was the protein-coding gene *USP16,* reported to regulate tumor development by modulating the proliferation and death of cancer cells [36,37]. According to our findings, USP16 methylation is increased in breast metastatic disease, in agreement with findings in hepatocellular carcinoma [36]. Overall, all the features of this signature are worthy of further attention in cancer biology studies.

The next model built can accurately perform in the early diagnosis of BrCa. Six equivalent five-feature signatures emerged, showing an AUC of 0.973 (0.920–1.000). Three features of the signatures, two lncRNAs and one lincRNA, have not been reported in cancer before. The gene feature LINC00623 has been correlated to the cisplatin response in ovarian cancer [38] and has also been associated with oral squamous cell carcinoma [39]. In addition, abnormal LINC00623 expression has also been correlated to poor survival of BrCa and kidney cancer patients [40]. Here, LINC00623 was found to be hypermethylated in stage-I BrCa in relation to normal breast tissue, suggesting an early methylation event in malignant transformation. Another feature of this signature, the protein coding gene AIM2, has been shown to suppress the development of multiple cancers [41]. AIM2 aberrant expression has been reported in several cancer types such as prostate cancer [42] and non-small-cell lung cancer [43]. Its suppressive and pre-apoptotic role in BrCa [44,45], implying a protective action, is in agreement with its hypomethylated status in early BrCa samples in our study. Protein coding *DNM2* was also included as a feature in this signature and was shown to be hypermethylated in Stage-I BrCa. DNM2 is known for its role in endocytic cell trafficking and microtubule dynamics. High *DNM2* expression is reported in bladder cancer [46], whereas Chernikova et al. showed that levels of *DNM2* could predict chemotherapy outcomes for triple-negative BrCa patients [47]. Another gene included in the signatures and found to be hypermethylated in early BrCa, *SSH1*, has been presented to be a cancer progression factor [48]. *SSH1* was reported to be highly expressed in gastric cancer and is correlated to poor clinical outcomes [48]. Its high expression in colon cancer was also associated with poor cancer parameters and non-treatment responses [49]. *PDGFRB*, detected as being hypermethylated in stage-I BrCa, had been reported to increase proliferation and migration in several cancers [50,51,52], *PDGFRB* expression was correlated with less favorable clinicopathological parameters and shorter survival in BrCa [22,53]. A highly studied gene, *TIMP3*, which plays a key role in cancer development and progression [54], was also found to be hypermethylated. TIMP3 plasma levels were significantly lower in oral cancer and were associated with tumor stage and size [55]. Hypermethylation of *TIMP3* was correlated to poor cancer parameters in BrCa [56], in bladder cancer [57] and in gastric cancer [58]. Finally, *AP2M1* was found to be hypermethylated in Stage-I BrCa tissues in relation to normal tissues. To the best of our knowledge, only one relevant study has addressed its value as a predictive biomarker in hepatocellular carcinoma, showing its correlation to prognosis [59].

To further elaborate on the functional role of the selected protein genes that were included in the signatures to BrCa pathophysiology, we used a computational tool for predicting functional associations amongst proteins, UniReD. This platform analyzes biomedical literature in order to extract published protein associations and to suggest undocumented ones. Notably, all the protein coding genes included in the JADBio produced signatures that were found to be associated with breast cancer pathways. Furthermore, when tested against 10 pre-selected genes with a well-established role in breast cancer, multiple associations were verified. This finding strengthens significantly our AutoML results because it validates selected features through a completely different approach, especially considering that in our workflow selection was performed in non-preselected features.

Along with further investigation for the involvement of the identified features in cancer biology, we believe that the three highly performing signatures are of great translational value and offer a mature starting point for diagnostic R&D. They provide the basis for targeted assays such as multiplex qPCR, ddPCR or NGS targeted panels to be validated in prospective clinical studies. Ideally, the emerged genes could be further studied in liquid biopsy material, offering minimally invasive choices, adding new prospects in terms of their clinical value. In addition to the two advantages discussed earlier regarding the translational potential of the biosignatures, i.e., a low feature number and accurate performance prediction, the production of equivalent signatures is also important, offering alternatives for clinical assay development.

As comparing methylomes from early and advanced BrCa did not result in powerful classifiers, we speculate that a lack of distinct methylation patterns developed during cancer progression. Similarly, in our cohort, methylation-based signatures could not predict survival in primary BrCa patients. In contrast, previous studies in tissue [60,61] or in liquid biopsies [4,9] have showed that specific gene methylation could serve as marker for survival prediction. Unfortunately, our metastatic cohort did not include adequate outcome data, allowing more powerful analysis. A lack of complete clinical information is common in the deposited -omics datasets, which undoubtfully raises an intrinsic limitation of data-driven approaches. Clearly, revisiting archived datasets gradually attracts more interest by researchers as new analyzing tools emerge. Thus, the quality and completeness of the data stored becomes a major issue and groups producing the original datasets are encouraged to share more information about their samples.

Supporting the role of aberrant methylation in breast carcinogenesis, differential analysis of methylomes identified 11,176 to 27,786 DMGs against different clinical end-points. Bioinformatic functional analysis identified regulation of transcription as a major biological process affected and the nucleus the main cellular component, whereas transcription factor activity and sequence-specific DNA binding were found to be highly involved molecular functions. This is not surprising, as a large number of oncogenic genes present abnormal expression as a result of altered regulation of transcription due to DNA methylation. In addition, methylation has the potential to modulate transcription factor activity and, vice versa, transcription factor DNA binding can also inhibit DNA methylation [62].

## 4. Materials and Methods

### 4.1. Data Sources

Raw DNA methylation data from BrCa and normal tissues and corresponding clinical data were obtained from TCGA (www.cancer.gov/tcga, accessed on 5 February 2020) and GEO (www.ncbi.nlm.nih.gov/geo, accessed on 10 February 2020) [63] databases. TCGA case inclusion criteria were: 1. Platform: Infinium Human Methylation 450 K bead-chipl 2. Primary site: breast; 3. Project: TCGA-BRCA; 4. Gender: female; 5. Age at diagnosis: 26–80 years; 6. Race: white, black or African American, Asian and not reported. A total of 730 cases were downloaded. The GEO database was searched using ‘Breast cancer’ and ‘Metastatic Breast cancer’ as keywords and ‘Methylation profiling by array’ as study type: 84 and 10 studies were found, respectively. Those using the Infinium Human Methylation 450 K bead-chip array and providing adequate raw and clinical data were selected for further analysis, i.e., five studies, namely GSE72245, GSE72251 [23], GSE88883 [64], GSE108576 [24] and GSE74214. Analysis was performed vs. five major clinically relevant end-points, as presented in Table 3. External validation of analysis comparing BrCa vs. normal patients was made in an external dataset generated from GSE66313 [65], GSE72254 [23] and GSE101445 [66] GEO studies, consisting of 98 BrCa tissues and 20 normal breast tissues. The study workflow is depicted in Figure 1**.**

### 4.2. Data Preprocessing and DNA Methylation Analysis

Raw DNA methylation data (IDAT files) and sample annotation files were subjected to the Bioconductor R package RnBeads v2.0 (rnbeads.org/index.html, accessed on 20 March 2020) [67]. RnBeads is a software tool suitable for large-scale analysis, interpretation and visualization of DNA methylation data. In our workflow, genes were chosen as the genomic region of interest and were analyzed for each of the five endpoints. In order to correct for technical variation in background fluorescence signal, the methylumi-noob method was used [68]. Beta-mixture quantile normalization (BMIQ) was used as a normalization method to adjust the beta-values of type-II design probes into a statistical distribution characteristic of type-I probes [69]. Subsequently, polymorphic probes or probes outside of the CpG context, as well as probes on sex chromosomes, were removed [70]. Probes/samples with the highest fraction of unreliable measurements were removed from further analysis using the greedycut algorithm. Consequently, normalized β(beta)-values for each gene were generated, representing the average methylated probe intensity divided by the overall intensity (the sum of methylated and unmethylated probe intensities), plus an offset of 100 [71]. DNA methylation differences were analyzed using hierarchical linear models as implemented in the limma package [72], provided by the RnBeads pipeline. Methylation β values are expressed as decimal values between 0.0 (no methylation) and 1.0 (full methylation). Differentially methylated genes (DMGs) were ranked based on the combined rank score which uses a combination of the change in mean methylation, the quotient of mean methylation and the false discovery rate (FDR)-adjusted *p*-value for further downstream analysis.

### 4.3. Functional Analysis of DMGs

The biological functions of the first 250 ranked DMGs were further investigated using publicly available tools. The Database for Annotation, Visualization and Integrated Discovery (DAVID) v6.8 (david.abcc.ncifcrf.gov, accessed on 4 May 2020) [73] was used for Gene Ontology (GO) analysis, dividing DMGs into three functional groups: Biological Process (BP), Cellular Component (CC) and Molecular Function (MF). DAVID provides a set of functional annotation tools to understand the biological meaning behind large list of genes. The Benjamini–Hochberg FDR < 0.05 was set as the cutoff criterion in GO analysis. In addition, we used ConsensusPathDB-Human Release 34 (cpdb.molgen.mpg.de, accessed on 10 May 2020) [74] to perform Kyoto Encyclopedia of Genes and Genomes (KEGG) and Reactome analysis. ConsensusPathDB integrates interaction networks in *Homo sapiens* including binary and complex protein–protein, genetic, metabolic, signaling, gene regulatory and drug–target interactions, as well as biochemical pathways. Finally, in order to evaluate the relationships among DMGs, we analyzed them using the Search Tool for the Retrieval of Interacting Genes (STRING) v11.0 (string-db.org, accessed on 20 June 2020) [75] and protein–protein interaction (PPI) networks were visualized by Cytoscape 3.8.2 (cytoscape.org/, accessed on 20 March 2021) [76].

### 4.4. Automated Machine Learning Analysis

The AutoML technology Just Add Data Bio (JADBio) (www.JADBio.com/, accessed on 25 September 2020) [14] was used to produce diagnostic and prognostic signatures/classifiers based on the β-values of the methylation data. JADBio is applicable to low-sample, high-dimensional -omics data and provides predictive models by employing standard, best-practice and state-of-the-art statistical and machine learning methods. JADBio automatically produces predictive models either for a discrete (classification), a continuous (regression) or a time-to-event (survival analysis) outcome. Specifically, JADBio [14] has the following functionality and properties: (a) given a 2D matrix of data, it automatically produces predictive models for a categorical (classification), continuous (regression), or time-to-event (survival analysis) outcome. No selection of appropriate algorithms to apply is necessary, nor is tuning of their hyper-parameter values. The available classification algorithms are: random forest classification, support vector machine (SVM) and ridge logistic regression and classification decision trees; (b) it identifies multiple equivalent biosignatures; and (c) it produces conservative predictive performance estimates and corresponding confidence intervals. It reliably processes up to hundreds of thousands of features and sample sizes as low as a couple of dozen. JADBio also employs the recently developed Bootstrap Bias Corrected CV (BBC-CV) protocol for tuning the hyper-parameters of algorithms, while estimating performance and adjusting for multiple tries. A description of the JADBio architecture can be found in Montesanto et al. [77].

For all datasets, the performance was evaluated via internal validation (BBC-CV within each dataset). An extensive tuning effort was used and sample datasets were automatically split into training and validation groups in a proportion of 70/30 using JADBio.

### 4.5. Analysis with UniReD

We employed UniProt Related Documents (UniReD) (bioinformatics.med.uoc.gr/unired/help.php, accessed on 14 December 2020) [19] in order to crosscheck our gene features of produced signatures, which appear in Table 1. UniReD is a tool for predicting functional associations among proteins, based on related articles. We tested associations of proteins identified as features in the emerged classifiers against 10 genes known for their significant implication in breast cancer biology (see gene list in Table S6). We ran a UniReD analysis for each protein and searched in the proposed list of proteins predicted by UniReD as to whether we could identify a protein from Table 1. When we could not find the human protein, we searched for homologs of the protein or we ran UniReD using the mouse ortholog and we conducted the same analysis. We used a simple scoring system to rank the proteins of Table 1, i.e., we assigned one point when we found the human protein and 0.5 points whenever we were able to find a homolog of the protein or the same protein after conducting the analysis using the mouse ortholog. We subsequently ran a UniReD analysis for each protein listed in Table 1 (except for proteins RWDD4 and BRINP2, for which UniReD could not provide any information) and we examined whether we could find any association with breast cancer pathways (UniReD uses the KEGG pathway analysis system).

### 4.6. Statistical Analysis

The Smirnov–Kolmogorov test was applied in order to check the normality of age distributions among groups. Student’s *t*-test was then used to compare mean age between groups. Statistical significance was set at *p*-values < 0.05. Statistical analysis was performed using the IBM SPSS Statistics 21 software (IBM Corp. 2010. IBM SPSS Statistics for Windows, Version 21.0. Armonk, NY, USA).

## 5. Conclusions

Revisiting the available BrCa methylomes with innovative AutoML tools produced three highly performing simple signatures of diagnostic/prognostic power, which can add value to the clinical management of BrCa. Through complete bioinformatic analysis, DNA methylation emerged as an important mechanism in BrCa, as a great number of DMGs were identified between studied groups, and gene transcription was the key biological process affected.

## Figures and Tables

**Table 1:** Genes included in the predictive signatures and their methylation status.

Analysis	Signature’s Genes	Gene Type	Description	Methylation Status
1. BrCa vs. Normal	AC104435.5	lncRNA	NA	hypomethylated *
	AC002550.1	lncRNA	Antisense to C16orf88	hypermethylated *
	AC078802.1	lncRNA	Antisense to ACTRT3	hypomethylated *
	AC124283.3	lncRNA	Sense intronic to FOXK2	hypermethylated *
	DND1P1	Pseudogene	DND microRNA-mediated repression inhibitor 1	hypomethylated *
2. Primary vs. Metastatic BrCa	AL139011.1	lncRNA	NA	hypermethylated ^$^
	AD000671.3	lncRNA	NA	hypermethylated ^$^
	USP16	Protein coding	Ubiquitin-Specific-Processing Protease 16	hypermethylated ^$^
3. Stage I BrCa vs. Normal	AIM2	Protein coding	Absent in Melanoma 2	hypomethylated *
	AL513008.1	lncRNA	NA	hypermethylated *
	AC004884.2	lncRNA	NA	hypomethylated *
	LINC01563	lincRNA	NA	hypermethylated *
	DNM2	Protein coding	Dynamin 2	hypermethylated *
	SSH1	Protein coding	Slingshot protein phosphatase 1	hypermethylated *
	PDGFRB	Protein coding	Platelet-derived growth factor receptor beta	hypermethylated *
	TIMP3	Protein coding	TIMP metallopeptidase inhibitor 3	hypermethylated *
	AP2M1	Protein coding	Adaptor related protein complex 2 subunit mu 1	hypermethylated *
	LINC00623	lincRNA	NA	hypermethylated *
4. Early vs. Advanced BrCa	SMARCAD1	Protein coding	SWI/SNF-related, matrix-associated actin-dependent regulator of chromatin, subfamily a, containing DEAD/H box 1	hypermethylated ^#^
	RWDD4	Protein coding	RWD domain containing 4	hypermethylated ^#^
	RPF2	Protein coding	Ribosome production factor 2 homolog	hypermethylated ^#^
	WDR11	Protein coding	WD repeat domain 11	hypomethylated ^#^
	SNHG25	lncRNA	Small nucleolar RNA host gene 25	hypermethylated ^#^
5. Overall Survival	AP005436.3	lncRNA	NA	NA
	DDN-AS1	lncRNA	NA	NA
	IL17RE	Protein coding	Interleukin 17 receptor E	NA
	XX-C2158C12.2	lincRNA	NA	NA
	AL355916.2	lncRNA	NA	NA
	LINC00824	lincRNA	NA	NA
	NET1	Protein coding	Neuroepithelial Cell Transforming 1	NA
	BRINP2	Protein coding	BMP/Retinoic Acid Inducible Neural Specific 2	NA

* in relation to normal, ^#^ in relation to early BrCa stage, ^$^ in relation to primary BrCa. Abbreviations: BrCa = Breast Cancer, lncRNA = long non-coding RNA, lincRNA = long intergenic non-protein coding RNA, NA = non-available.

**Table 2:** Ranking of proteins of which the genes were included in the built classifying signatures according to their association to 10 genes known to be implicated in BrCa biology after analysis with UniReD.

Protein Name	Uniprot ID	UniReD Score
TIMP3	P35625	10
PDGFRB	P09619	8
DNM2	P50570	7.5
USP16	Q9Y5T5	7.5
NET1	Q7Z628	6.5
AIM2	O14862	6.5
WDR11	Q9BZH6	4
SMARCAD1	Q9H4L7	4
SSH1	Q8WYL5	3.5
AP2M1	Q96CW1	3.5
IL17RE	Q8NFR9	3
RPF2	Q9H7B2	2

**Table 3:** Comparisons, end-points, study group characteristics and clinical significance of the datasets used in the bioinformatic analysis.

Comparison	Clinical End-Point	Tissues	Age (years)	Stage	Significance
BrCa vs. Normal	BrCa disease	520 BrCa (primary and metastatic breast tumor)	49 (26–80)	102 Stage I 264 Stage II 114 Stage III 40 Stage IV	Diagnosis
		185 Normal breast	47 (26–80)	NA	
Primary vs. Metastatic BrCa	Metastasis	132 Primary BrCa	55 (47–55)	22 Stage I 75 Stage II 35 Stage III	Classification
		31 Metastatic BrCa	54 (41–80)	31 Stage IV	
Stage-I BrCa vs. Normal	Early disease	136 Stage I BrCa	54 (27–80)	136 Stage I	Early diagnosis
		111 Normal breast	58 (29–80)	NA	
Early vs. Advanced BrCa	Advanced disease	521 Early BrCa	58 (26–80)	115 Stage I 406 Stage II	Classification
		221 Advanced BrCa	55 (27–80)	221 Stage III	
Overall Survival	Survival	894 Primary BrCa	58 (26–80)	254 Stage I 375 Stage II 265 Stage III	Prognosis

Abbreviations: BrCa = breast cancer, NA = non-available.

## Data Availability

All produced data of this study are available upon reasonable request.

## Supplementary Materials

The following are available online at https://www.mdpi.com/article/10.3390/cancers13071677/s1, Figure S1 (A) The significantly enriched pathways of the top 250 DMGs based on KEGG and REACTOME pathway analysis. (B) Principal component analysis (PCA) plot presenting the separation between normal (blue) and BrCa tissues (green) within the training group. (C) Uniform manifold approximation and projection (UMAP) plot showing successful discrimination between normal (blue) and BrCa tissues (green) within the training group. (D) PCA plot of validation group verifying the discrimination power of the model. Abbreviations: BrCa = breast cancer, DMGs = differentially methylated genes, PCA = principal component analysis, UMAP = uniform manifold approximation and projection. Figure S2 (A) PCA plot presenting excellent separation between primary BrCa (blue) and metastatic BrCa (green) of the training group. (B) UMAP plot showing discrimination between primary BrCa (blue) and metastatic BrCa (green) of the training group. (C) PCA plot of validation group verifying the stability of the model. Abbreviations: BrCa = breast cancer, DMGs = differentially methylated genes, PCA = principal component analysis, UMAP = uniform manifold approximation and projection. Figure S3 (A) The significantly enriched pathways of the top 250 DMGs based on KEGG and REACTOME pathway analysis. (B) PCA plot presenting successful separation between normal (blue) and Stage-I BrCa tissues (green) of the training group. (C) UMAP plot showing discrimination between normal (blue) and Stage-I BrCa tissues (green) of the training group. (D) PCA plot of the validation group verifying the accuracy of the model. Abbreviations: BrCa = breast cancer, DMGs = differentially methylated genes, PCA = principal component analysis, UMAP = uniform manifold approximation and projection. Figure S4 A. PCA plot presenting poor separation between early- (blue) and advanced-stage BrCa (green) in the training group. (B) UMAP plot presenting poor discrimination between early- (blue) and advanced- stage BrCa (green) in the training group. Abbreviations: BrCa = breast cancer, DMGs = differentially methylated genes, PCA = principal component analysis, UMAP = uniform manifold approximation and projection. Table S1: List of the 250 top-ranking differentially methylated genes between BrCa and normal tissues. Table S2: Genes counts and enrichment percentages provided by DAVID tool between BrCa and normal breast tissues. Table S3: List of the 250 top-ranking differentially methylated genes between primary and metastatic BrCa tissues. Table S4: List of the 250 top-ranking differentially methylated genes between Stage I BrCa and normal tissues. Table S5: Gene counts and enrichment percentages provided by DAVID tool between Stage-I BrCa and normal breast tissues. Table S6: Genes involved in the pathophysiology of breast cancer according to the literature. File S1: Protein–protein interaction (PPI) network visualization by Cytoscape concerning the 250 top-ranking differentially methylated genes comparing BrCa and normal tissues. File S2: PPI network visualization by Cytoscape concerning the 250 top-ranking differentially methylated genes comparing primary and metastatic BrCa tissues. File S3: PPI network visualization by Cytoscape concerning the 250 top-ranking differentially methylated genes comparing Stage-I BrCa and normal tissues. File S4: PPI network visualization by Cytoscape concerning the 250 top-ranking differentially methylated genes comparing early-stage BrCa vs. advanced-stage BrCa tissues.
