# Supplementary material for: Deciphering the Methylation Landscape in Breast Cancer: Diagnostic and Prognostic Biosignatures through Automated Machine Learning
Source: Cancers (Basel). 2021 Apr 2;13(7):1677. doi: 10.3390/cancers13071677 (PMC8037759; doi:10.3390/cancers13071677)
Supplement: Supplementary file 1 [file cancers-13-01677-s001.zip › Supplementary Tables S1-S6.docx]

**Supplementary Table 1 List of the 250 top-ranking differentially methylated genes between BrCa and normal tissues.**

| **Symbol** | **Mean Methylation difference** | **FDR** | **Methylation status in BrCa in relation to normal** |
| --- | --- | --- | --- |
| OR2M7 | -0,3963 | 2,20E-85 | Hypomethylated |
| OR2M3 | -0,3475 | 8,44E-73 | Hypomethylated |
| CCL11 | -0,3449 | 7,59E-100 | Hypomethylated |
| FGF12-AS1 | -0,3355 | 5,26E-77 | Hypomethylated |
| MIR21 | -0,2739 | 3,88E-92 | Hypomethylated |
| BPIFA4P | -0,2386 | 6,13E-71 | Hypomethylated |
| BRWD1-AS1 | -0,2229 | 1,33E-55 | Hypomethylated |
| LILRA2 | -0,2213 | 1,43E-54 | Hypomethylated |
| TSTD1 | -0,2129 | 8,44E-89 | Hypomethylated |
| SNORD52 | -0,1561 | 4,14E-50 | Hypomethylated |
| CD34 | 0,1531 | 7,09E-66 | Hypermethylated |
| EDNRB | 0,1531 | 3,78E-53 | Hypermethylated |
| CR1 | 0,1536 | 2,58E-56 | Hypermethylated |
| NEFL | 0,1540 | 2,99E-66 | Hypermethylated |
| BHMT2 | 0,1540 | 3,18E-48 | Hypermethylated |
| NKX2-1-AS1 | 0,1548 | 4,58E-46 | Hypermethylated |
| SNCA | 0,1551 | 1,53E-46 | Hypermethylated |
| ITPRIPL1 | 0,1553 | 3,82E-60 | Hypermethylated |
| HNF1B | 0,1560 | 1,70E-44 | Hypermethylated |
| SYNDIG1 | 0,1567 | 1,48E-50 | Hypermethylated |
| GAL3ST3 | 0,1569 | 4,73E-52 | Hypermethylated |
| FOXB2 | 0,1570 | 1,71E-50 | Hypermethylated |
| RCN1 | 0,1572 | 6,96E-48 | Hypermethylated |
| AKR1B1 | 0,1572 | 2,81E-46 | Hypermethylated |
| CEBPA-AS1 | 0,1573 | 1,93E-46 | Hypermethylated |
| LINC01158 | 0,1576 | 6,88E-52 | Hypermethylated |
| RAX | 0,1577 | 3,40E-48 | Hypermethylated |
| MIR9-3 | 0,1579 | 6,73E-50 | Hypermethylated |
| ERN2 | 0,1581 | 3,25E-73 | Hypermethylated |
| ST8SIA3 | 0,1584 | 4,47E-70 | Hypermethylated |
| GRIA4 | 0,1604 | 7,15E-52 | Hypermethylated |
| SALL2 | 0,1606 | 9,22E-68 | Hypermethylated |
| GSC2 | 0,1610 | 3,67E-47 | Hypermethylated |
| PCDH17 | 0,1611 | 1,27E-71 | Hypermethylated |
| EPHA5 | 0,1612 | 7,49E-49 | Hypermethylated |
| CYP2T2P | 0,1623 | 1,81E-57 | Hypermethylated |
| NEXN | 0,1624 | 1,48E-50 | Hypermethylated |
| TVP23BP1 | 0,1624 | 1,92E-70 | Hypermethylated |
| UGT3A2 | 0,1626 | 7,61E-56 | Hypermethylated |
| AKNA | 0,1626 | 1,75E-51 | Hypermethylated |
| ZNF559-ZNF177 | 0,1636 | 3,19E-66 | Hypermethylated |
| HOXD9 | 0,1636 | 1,72E-48 | Hypermethylated |
| ZNF232 | 0,1638 | 1,87E-59 | Hypermethylated |
| CA3 | 0,1658 | 1,24E-62 | Hypermethylated |
| RXFP3 | 0,1671 | 8,56E-48 | Hypermethylated |
| KCNE3 | 0,1674 | 4,64E-58 | Hypermethylated |
| MEOX2 | 0,1691 | 1,65E-48 | Hypermethylated |
| NR2E1 | 0,1692 | 5,00E-45 | Hypermethylated |
| VGLL2 | 0,1694 | 3,91E-45 | Hypermethylated |
| ULK4P2 | 0,1699 | 1,99E-56 | Hypermethylated |
| GNG11 | 0,1704 | 1,86E-46 | Hypermethylated |
| CHST2 | 0,1711 | 5,04E-50 | Hypermethylated |
| GPR149 | 0,1716 | 6,18E-49 | Hypermethylated |
| ADAMTS20 | 0,1719 | 8,85E-62 | Hypermethylated |
| LHFPL4 | 0,1724 | 1,22E-70 | Hypermethylated |
| UBA7 | 0,1728 | 2,77E-53 | Hypermethylated |
| ATOH1 | 0,1731 | 1,72E-44 | Hypermethylated |
| UNCX | 0,1733 | 1,04E-54 | Hypermethylated |
| CHL1-AS2 | 0,1736 | 7,16E-49 | Hypermethylated |
| CCDC67 | 0,1744 | 1,33E-48 | Hypermethylated |
| KCNAB3 | 0,1754 | 4,71E-50 | Hypermethylated |
| CCDC8 | 0,1759 | 2,68E-47 | Hypermethylated |
| PCSK1 | 0,1768 | 4,53E-62 | Hypermethylated |
| DLG5-AS1 | 0,1768 | 1,50E-65 | Hypermethylated |
| MGARP | 0,1778 | 8,67E-49 | Hypermethylated |
| GABRA4 | 0,1785 | 3,76E-78 | Hypermethylated |
| KLHL1 | 0,1789 | 1,98E-59 | Hypermethylated |
| ADRB3 | 0,1806 | 2,73E-47 | Hypermethylated |
| TTYH1 | 0,1811 | 2,09E-48 | Hypermethylated |
| MOS | 0,1815 | 2,12E-55 | Hypermethylated |
| HTR1A | 0,1818 | 5,30E-59 | Hypermethylated |
| INA | 0,1821 | 2,72E-61 | Hypermethylated |
| RBM27 | 0,1829 | 9,84E-56 | Hypermethylated |
| CRCT1 | 0,1841 | 1,22E-47 | Hypermethylated |
| CLEC11A | 0,1843 | 1,26E-49 | Hypermethylated |
| OTP | 0,1844 | 5,33E-55 | Hypermethylated |
| OLIG2 | 0,1846 | 3,02E-48 | Hypermethylated |
| VSX1 | 0,1847 | 1,18E-58 | Hypermethylated |
| DGAT2L7P | 0,1849 | 1,03E-48 | Hypermethylated |
| PRSS30P | 0,1851 | 7,53E-59 | Hypermethylated |
| ZFHX4-AS1 | 0,1854 | 5,82E-50 | Hypermethylated |
| FSIP2 | 0,1855 | 1,71E-47 | Hypermethylated |
| ZNF177 | 0,1860 | 2,56E-75 | Hypermethylated |
| LHX9 | 0,1863 | 2,90E-46 | Hypermethylated |
| CD46P1 | 0,1873 | 1,21E-44 | Hypermethylated |
| ZNF571-AS1 | 0,1878 | 7,91E-78 | Hypermethylated |
| OLIG3 | 0,1879 | 5,24E-52 | Hypermethylated |
| KCTD8 | 0,1881 | 1,37E-56 | Hypermethylated |
| HIST2H3D | 0,1900 | 1,09E-74 | Hypermethylated |
| USP44 | 0,1905 | 8,19E-59 | Hypermethylated |
| GRASP | 0,1907 | 5,50E-83 | Hypermethylated |
| NPY | 0,1914 | 2,17E-64 | Hypermethylated |
| MSX2P1 | 0,1917 | 2,12E-56 | Hypermethylated |
| POU3F3 | 0,1920 | 3,04E-45 | Hypermethylated |
| PCDH10 | 0,1922 | 1,32E-44 | Hypermethylated |
| ZNF560 | 0,1931 | 3,50E-86 | Hypermethylated |
| WT1-AS | 0,1936 | 1,75E-47 | Hypermethylated |
| QRFPR | 0,1943 | 1,35E-45 | Hypermethylated |
| L1TD1 | 0,1944 | 2,65E-72 | Hypermethylated |
| ACTL6B | 0,1947 | 7,13E-71 | Hypermethylated |
| DNASE1L2 | 0,1947 | 8,49E-57 | Hypermethylated |
| MSC | 0,1954 | 2,65E-52 | Hypermethylated |
| SERPING1 | 0,1955 | 2,02E-76 | Hypermethylated |
| YWHAEP7 | 0,1956 | 4,61E-45 | Hypermethylated |
| PRDM13 | 0,1957 | 4,83E-65 | Hypermethylated |
| SLITRK1 | 0,1965 | 1,75E-51 | Hypermethylated |
| FEZF2 | 0,1975 | 1,89E-56 | Hypermethylated |
| NPBWR1 | 0,1975 | 1,24E-53 | Hypermethylated |
| NKX2-1 | 0,1980 | 2,12E-58 | Hypermethylated |
| OTX2 | 0,1989 | 9,33E-66 | Hypermethylated |
| FZD7 | 0,1991 | 5,51E-49 | Hypermethylated |
| PRLHR | 0,1996 | 1,45E-78 | Hypermethylated |
| FAM162B | 0,1996 | 6,56E-49 | Hypermethylated |
| LHX8 | 0,1998 | 5,21E-49 | Hypermethylated |
| ENPP2 | 0,2004 | 3,97E-68 | Hypermethylated |
| PTF1A | 0,2010 | 8,84E-67 | Hypermethylated |
| FAM90A28P | 0,2021 | 1,02E-49 | Hypermethylated |
| ZNF551 | 0,2021 | 4,98E-48 | Hypermethylated |
| TBX15 | 0,2024 | 1,33E-62 | Hypermethylated |
| EVX2 | 0,2024 | 7,51E-50 | Hypermethylated |
| GALR3 | 0,2027 | 1,10E-52 | Hypermethylated |
| ONECUT2 | 0,2031 | 5,41E-66 | Hypermethylated |
| CYP2A7P1 | 0,2033 | 5,63E-46 | Hypermethylated |
| HOXD-AS2 | 0,2042 | 3,91E-57 | Hypermethylated |
| AVPR1A | 0,2043 | 2,92E-68 | Hypermethylated |
| PDX1 | 0,2047 | 4,44E-61 | Hypermethylated |
| CDX2 | 0,2052 | 2,29E-62 | Hypermethylated |
| TLX2 | 0,2053 | 1,37E-53 | Hypermethylated |
| TFAP2D | 0,2059 | 1,47E-53 | Hypermethylated |
| ZIC1 | 0,2061 | 3,72E-50 | Hypermethylated |
| LHX1 | 0,2073 | 2,68E-51 | Hypermethylated |
| ZIC4 | 0,2078 | 5,36E-57 | Hypermethylated |
| SOX14 | 0,2086 | 3,82E-69 | Hypermethylated |
| GHSR | 0,2087 | 1,19E-92 | Hypermethylated |
| GALR1 | 0,2088 | 5,58E-51 | Hypermethylated |
| DMRTA2 | 0,2091 | 7,79E-61 | Hypermethylated |
| SLC32A1 | 0,2095 | 8,58E-69 | Hypermethylated |
| NEUROG3 | 0,2102 | 1,68E-71 | Hypermethylated |
| FOXQ1 | 0,2106 | 6,26E-52 | Hypermethylated |
| HOXA4 | 0,2111 | 9,23E-56 | Hypermethylated |
| FOXA2 | 0,2119 | 1,99E-64 | Hypermethylated |
| MARCH11 | 0,2125 | 8,40E-52 | Hypermethylated |
| VSTM2B | 0,2125 | 1,49E-67 | Hypermethylated |
| CHRND | 0,2126 | 8,15E-65 | Hypermethylated |
| CYP2A13 | 0,2129 | 1,46E-72 | Hypermethylated |
| CARTPT | 0,2133 | 2,14E-57 | Hypermethylated |
| ACTA1 | 0,2143 | 4,63E-72 | Hypermethylated |
| C12orf68 | 0,2144 | 1,43E-88 | Hypermethylated |
| YBX3P1 | 0,2155 | 2,74E-74 | Hypermethylated |
| RESP18 | 0,2159 | 5,93E-75 | Hypermethylated |
| WT1 | 0,2185 | 1,37E-62 | Hypermethylated |
| LINC00966 | 0,2191 | 7,40E-56 | Hypermethylated |
| HSPB6 | 0,2191 | 9,23E-59 | Hypermethylated |
| PHOX2B | 0,2194 | 4,44E-57 | Hypermethylated |
| NETO1 | 0,2217 | 4,82E-89 | Hypermethylated |
| MAGI2-AS3 | 0,2219 | 4,23E-76 | Hypermethylated |
| C6orf58 | 0,2221 | 5,81E-61 | Hypermethylated |
| ZNF876P | 0,2224 | 1,26E-74 | Hypermethylated |
| EID3 | 0,2228 | 4,54E-48 | Hypermethylated |
| TCF24 | 0,2233 | 5,12E-63 | Hypermethylated |
| PCDH8 | 0,2237 | 7,60E-62 | Hypermethylated |
| SSTR1 | 0,2240 | 3,61E-68 | Hypermethylated |
| MIR663A | 0,2250 | 1,68E-89 | Hypermethylated |
| SFTA3 | 0,2263 | 3,05E-69 | Hypermethylated |
| C14orf39 | 0,2271 | 6,43E-65 | Hypermethylated |
| BHLHA9 | 0,2291 | 3,20E-52 | Hypermethylated |
| SST | 0,2300 | 1,68E-82 | Hypermethylated |
| NEFH | 0,2304 | 1,23E-86 | Hypermethylated |
| NRIP2 | 0,2312 | 9,03E-49 | Hypermethylated |
| SOX1 | 0,2315 | 2,69E-78 | Hypermethylated |
| NMBR | 0,2328 | 3,59E-77 | Hypermethylated |
| HMX2 | 0,2333 | 6,92E-63 | Hypermethylated |
| SCGB1B2P | 0,2338 | 1,09E-78 | Hypermethylated |
| GLIPR1L1 | 0,2339 | 1,16E-63 | Hypermethylated |
| FOXD4 | 0,2341 | 4,57E-78 | Hypermethylated |
| ALX1 | 0,2343 | 5,14E-61 | Hypermethylated |
| UNC93B7 | 0,2371 | 1,01E-88 | Hypermethylated |
| POU4F3 | 0,2380 | 6,10E-76 | Hypermethylated |
| CDO1 | 0,2386 | 1,33E-74 | Hypermethylated |
| NES | 0,2395 | 1,21E-65 | Hypermethylated |
| NKX2-6 | 0,2422 | 2,07E-87 | Hypermethylated |
| BARHL2 | 0,2428 | 2,81E-74 | Hypermethylated |
| NKX2-2 | 0,2431 | 1,44E-54 | Hypermethylated |
| SPARC | 0,2434 | 7,21E-78 | Hypermethylated |
| NEFM | 0,2438 | 3,41E-105 | Hypermethylated |
| HTR1B | 0,2452 | 1,12E-89 | Hypermethylated |
| DBX1 | 0,2454 | 4,09E-72 | Hypermethylated |
| TBX18 | 0,2454 | 1,56E-75 | Hypermethylated |
| CCDC36 | 0,2461 | 8,07E-81 | Hypermethylated |
| DSC3 | 0,2461 | 1,22E-61 | Hypermethylated |
| HOXD8 | 0,2462 | 8,67E-45 | Hypermethylated |
| RHOQP2 | 0,2479 | 2,64E-111 | Hypermethylated |
| HIST3H2BA | 0,2485 | 5,10E-63 | Hypermethylated |
| DRD5 | 0,2487 | 9,44E-72 | Hypermethylated |
| PRAP1 | 0,2508 | 4,32E-64 | Hypermethylated |
| FOXG1 | 0,2523 | 6,59E-67 | Hypermethylated |
| ABCB10P4 | 0,2533 | 1,70E-109 | Hypermethylated |
| POU4F1 | 0,2541 | 2,64E-60 | Hypermethylated |
| HOXD12 | 0,2545 | 4,54E-89 | Hypermethylated |
| SIX6 | 0,2556 | 1,67E-67 | Hypermethylated |
| HOTAIRM1 | 0,2560 | 1,22E-71 | Hypermethylated |
| NXPH1 | 0,2562 | 6,81E-81 | Hypermethylated |
| NKX2-4 | 0,2565 | 9,06E-86 | Hypermethylated |
| FOXE3 | 0,2565 | 3,91E-81 | Hypermethylated |
| RNU5F-2P | 0,2574 | 1,96E-51 | Hypermethylated |
| SLC18A3 | 0,2587 | 4,43E-105 | Hypermethylated |
| SKOR2 | 0,2589 | 4,48E-122 | Hypermethylated |
| NKX1-1 | 0,2595 | 9,12E-86 | Hypermethylated |
| MIR9-1 | 0,2612 | 3,05E-56 | Hypermethylated |
| HCG16 | 0,2618 | 8,00E-56 | Hypermethylated |
| ASCL4 | 0,2631 | 2,66E-59 | Hypermethylated |
| NKAPL | 0,2647 | 3,24E-88 | Hypermethylated |
| CPXM1 | 0,2651 | 8,58E-76 | Hypermethylated |
| NKX6-2 | 0,2666 | 5,96E-99 | Hypermethylated |
| SOX17 | 0,2688 | 1,01E-89 | Hypermethylated |
| SYNE1-AS1 | 0,2698 | 2,10E-67 | Hypermethylated |
| ZNF728 | 0,2728 | 2,29E-92 | Hypermethylated |
| UTF1 | 0,2732 | 1,80E-71 | Hypermethylated |
| BHLHE23 | 0,2747 | 1,88E-81 | Hypermethylated |
| MED15P3 | 0,2757 | 6,82E-59 | Hypermethylated |
| KCNJ2-AS1 | 0,2759 | 8,94E-74 | Hypermethylated |
| GABRG2 | 0,2762 | 7,47E-79 | Hypermethylated |
| ATXN8OS | 0,2768 | 1,33E-81 | Hypermethylated |
| NEUROD1 | 0,2768 | 5,72E-84 | Hypermethylated |
| OXT | 0,2782 | 3,38E-66 | Hypermethylated |
| IFNL4P1 | 0,2810 | 4,09E-86 | Hypermethylated |
| CLEC14A | 0,2837 | 9,26E-87 | Hypermethylated |
| GSX1 | 0,2892 | 4,20E-90 | Hypermethylated |
| HIST1H4F | 0,2933 | 2,14E-64 | Hypermethylated |
| COX11P1 | 0,2950 | 1,86E-95 | Hypermethylated |
| HIST2H3PS2 | 0,2967 | 2,35E-152 | Hypermethylated |
| POU4F2 | 0,2970 | 6,84E-105 | Hypermethylated |
| OR2I1P | 0,2975 | 2,95E-78 | Hypermethylated |
| FOXD3 | 0,2987 | 8,84E-70 | Hypermethylated |
| MIR129-2 | 0,3034 | 4,45E-75 | Hypermethylated |
| BCHE | 0,3047 | 8,89E-53 | Hypermethylated |
| TAC1 | 0,3067 | 2,02E-85 | Hypermethylated |
| HIST1H1A | 0,3122 | 6,56E-51 | Hypermethylated |
| ANXA1 | 0,3133 | 2,06E-45 | Hypermethylated |
| NKX2-2-AS1 | 0,3154 | 1,38E-67 | Hypermethylated |
| MT1L | 0,3172 | 5,89E-49 | Hypermethylated |
| TRH | 0,3184 | 3,40E-107 | Hypermethylated |
| CCDC181 | 0,3222 | 1,76E-100 | Hypermethylated |
| ZNF154 | 0,3232 | 3,79E-62 | Hypermethylated |
| PRAC1 | 0,3242 | 2,09E-59 | Hypermethylated |
| ZPBP2 | 0,3262 | 4,21E-116 | Hypermethylated |
| PDIA3P1 | 0,3274 | 1,54E-92 | Hypermethylated |
| FERD3L | 0,3516 | 8,07E-109 | Hypermethylated |
| MIR124-2 | 0,3782 | 4,29E-84 | Hypermethylated |
| COX7A1 | 0,3988 | 5,19E-143 | Hypermethylated |

**Supplementary Table 2 Genes counts and enrichment percent provided by DAVID tool between BrCa**

**and normal breast tissues**

.

| **Biological Process** | **Genes count** | **% Enrichment** | **P-Value** | **Benjamini** |
| --- | --- | --- | --- | --- |
| transcription from RNA polymerase II promoter | 28 | 11,4 | 1,90E-11 | 2,20E-08 |
| positive regulation of transcription from RNA polymerase II promoter | 38 | 15,4 | 4,60E-11 | 2,70E-08 |
| negative regulation of transcription from RNA polymerase II promoter | 31 | 12,6 | 4,00E-10 | 1,60E-07 |
| endocrine pancreas development | 8 | 3,3 | 1,00E-08 | 3,10E-06 |
| regulation of transcription, DNA-templated | 42 | 17,1 | 5,40E-08 | 1,30E-05 |
| transcription, DNA-templated | 49 | 19,9 | 7,80E-08 | 1,50E-05 |
| neuropeptide signaling pathway | 11 | 4,5 | 1,80E-07 | 3,10E-05 |
| chemical synaptic transmission | 14 | 5,7 | 3,00E-06 | 4,40E-04 |
| G-protein coupled receptor signaling pathway, coupled to cyclic nucleotide second messenger | 7 | 2,8 | 1,10E-05 | 1,50E-03 |
| pattern specification process | 5 | 2 | 2,50E-04 | 2,90E-02 |
| positive regulation of renal sodium excretion | 4 | 1,6 | 2,80E-04 | 3,00E-02 |
| neurofilament bundle assembly | 3 | 1,2 | 3,70E-04 | 3,60E-02 |
| regulation of transcription from RNA polymerase II promoter | 15 | 6,1 | 4,30E-04 | 3,90E-02 |
| **Cellular Component** | **Genes Count** | **% Enrichment** | **P-Value** | **Benjamini** |
| nucleus | 89 | 36,2 | 9,40E-06 | 1,60E-03 |
| neurofilament | 4 | 1,6 | 6,90E-05 | 6,10E-03 |
| **Molecular Function** | **Genes Count** | **% Enrichment** | **P-Value** | **Benjamini** |
| sequence-specific DNA binding | 41 | 16,7 | 7,90E-23 | 2,00E-20 |
| transcription factor activity, sequence-specific DNA binding | 38 | 15,4 | 1,90E-11 | 2,40E-09 |
| transcriptional activator activity, RNA polymerase II core promoter proximal region sequence-specific binding | 19 | 7,7 | 1,30E-10 | 1,10E-08 |
| RNA polymerase II core promoter proximal region sequence-specific DNA binding | 21 | 8,5 | 2,50E-09 | 1,60E-07 |
| RNA polymerase II regulatory region sequence-specific DNA binding | 13 | 5,3 | 3,40E-06 | 1,80E-04 |
| RNA polymerase II transcription factor activity, sequence-specific DNA binding | 11 | 4,5 | 2,00E-05 | 8,80E-04 |
| transcription factor activity, RNA polymerase II distal enhancer sequence-specific binding | 7 | 2,8 | 8,70E-05 | 3,20E-03 |
| peptide hormone binding | 5 | 2 | 2,10E-04 | 6,70E-03 |
| protein dimerization activity | 9 | 3,7 | 2,60E-04 | 7,50E-03 |
| neuropeptide binding | 4 | 1,6 | 1,30E-03 | 3,40E-02 |

**Supplementary Table 3 List of the 250 top-ranking differentially methylated genes between primary and metastatic BrCa tissues.**

| **Symbol** | **Mean Methylation difference** | **FDR** | **Methylation status in metastatic BrCa in relation to primary BrCa** |
| --- | --- | --- | --- |
| OVCH2 | 0,3027 | 1,20E-09 | hypermethylated |
| PRAC1 | 0,2367 | 1,77E-08 | hypermethylated |
| MTND5P28 | 0,1864 | 3,18E-11 | hypermethylated |
| GP1BB | 0,1701 | 2,50E-09 | hypermethylated |
| OXT | 0,1673 | 1,15E-09 | hypermethylated |
| HCG20 | 0,1633 | 1,78E-09 | hypermethylated |
| MIR3655 | 0,1191 | 9,82E-19 | hypermethylated |
| CHMP4A | 0,1130 | 9,31E-11 | hypermethylated |
| ZNF852 | 0,1101 | 3,89E-36 | hypermethylated |
| MIR933 | 0,1043 | 6,85E-23 | hypermethylated |
| XRCC6BP1 | 0,1002 | 1,03E-18 | hypermethylated |
| ATG4C | 0,0985 | 1,48E-13 | hypermethylated |
| ZNF567 | 0,0983 | 2,87E-18 | hypermethylated |
| ITGA5 | 0,0913 | 6,99E-12 | hypermethylated |
| XBP1 | 0,0892 | 2,71E-24 | hypermethylated |
| DECR1 | 0,0859 | 4,71E-12 | hypermethylated |
| LINC01063 | 0,0851 | 3,02E-19 | hypermethylated |
| APOL1 | 0,0838 | 6,23E-11 | hypermethylated |
| TFCP2 | 0,0832 | 3,48E-11 | hypermethylated |
| RARRES3 | 0,0824 | 1,51E-13 | hypermethylated |
| DAXX | 0,0812 | 2,60E-09 | hypermethylated |
| OTX1 | 0,0803 | 5,19E-12 | hypermethylated |
| IER3 | 0,0790 | 1,28E-19 | hypermethylated |
| ZNF571 | 0,0786 | 1,18E-26 | hypermethylated |
| SEPT5 | 0,0769 | 1,68E-08 | hypermethylated |
| TIGD3 | 0,0765 | 1,71E-09 | hypermethylated |
| RPS27 | 0,0765 | 1,89E-24 | hypermethylated |
| SP100 | 0,0759 | 4,27E-20 | hypermethylated |
| SNORD42B | 0,0758 | 4,14E-20 | hypermethylated |
| COX6A1 | 0,0757 | 3,35E-14 | hypermethylated |
| GNPDA2 | 0,0751 | 5,14E-12 | hypermethylated |
| APOBEC3B | 0,0751 | 7,70E-13 | hypermethylated |
| MIR330 | 0,0750 | 5,50E-12 | hypermethylated |
| CD27-AS1 | 0,0702 | 4,44E-11 | hypermethylated |
| IL10RB | 0,0686 | 1,03E-17 | hypermethylated |
| CAAP1 | 0,0676 | 3,95E-32 | hypermethylated |
| C19orf82 | 0,0660 | 4,06E-10 | hypermethylated |
| NNT | 0,0655 | 6,09E-10 | hypermethylated |
| MIR4710 | 0,0654 | 4,65E-14 | hypermethylated |
| ZFP36L2 | 0,0651 | 6,40E-09 | hypermethylated |
| HIST1H2AL | 0,0641 | 1,47E-10 | hypermethylated |
| ZNF585A | 0,0641 | 3,75E-22 | hypermethylated |
| OASL | 0,0627 | 1,50E-10 | hypermethylated |
| TPM2 | 0,0616 | 4,37E-14 | hypermethylated |
| SPG20 | 0,0611 | 7,41E-09 | hypermethylated |
| RAD51C | 0,0605 | 1,27E-12 | hypermethylated |
| UQCRB | 0,0593 | 1,68E-14 | hypermethylated |
| C10orf32-ASMT | 0,0592 | 8,66E-09 | hypermethylated |
| ZNF439 | 0,0589 | 3,70E-11 | hypermethylated |
| IQCJ-SCHIP1-AS1 | 0,0587 | 1,49E-17 | hypermethylated |
| SMG1P1 | 0,0587 | 5,24E-19 | hypermethylated |
| ADC | 0,0586 | 2,53E-17 | hypermethylated |
| DOCK9-AS2 | 0,0572 | 5,22E-17 | hypermethylated |
| MED20 | 0,0570 | 4,14E-16 | hypermethylated |
| XAF1 | 0,0565 | 1,25E-08 | hypermethylated |
| CASP8 | 0,0557 | 2,24E-12 | hypermethylated |
| UCHL3 | 0,0555 | 9,30E-19 | hypermethylated |
| FAM161A | 0,0554 | 5,99E-25 | hypermethylated |
| NR3C1 | 0,0547 | 2,07E-08 | hypermethylated |
| RPGRIP1 | 0,0544 | 1,01E-08 | hypermethylated |
| NCAPH2 | 0,0543 | 6,39E-10 | hypermethylated |
| TMEM106A | 0,0543 | 9,97E-18 | hypermethylated |
| EMC3-AS1 | 0,0542 | 2,55E-20 | hypermethylated |
| PYROXD1 | 0,0540 | 4,15E-13 | hypermethylated |
| ETAA1 | 0,0539 | 6,91E-15 | hypermethylated |
| CFL1 | 0,0539 | 4,33E-10 | hypermethylated |
| PPP1R18 | 0,0537 | 1,03E-08 | hypermethylated |
| TAGLN2 | 0,0537 | 1,67E-12 | hypermethylated |
| ZNF267 | 0,0532 | 1,07E-10 | hypermethylated |
| ADAMTSL4-AS1 | 0,0531 | 1,87E-30 | hypermethylated |
| LTC4S | 0,0527 | 5,58E-10 | hypermethylated |
| VPS13A-AS1 | 0,0522 | 3,34E-36 | hypermethylated |
| TRMT12 | 0,0521 | 8,35E-12 | hypermethylated |
| MANEA | 0,0516 | 1,59E-12 | hypermethylated |
| C19orf83 | 0,0516 | 4,58E-14 | hypermethylated |
| PHF11 | 0,0506 | 4,77E-09 | hypermethylated |
| ZFP69B | 0,0506 | 2,66E-16 | hypermethylated |
| RAB14 | 0,0505 | 1,08E-16 | hypermethylated |
| RAB8B | 0,0505 | 7,38E-10 | hypermethylated |
| C19orf25 | 0,0504 | 3,15E-09 | hypermethylated |
| ZFP36L1 | 0,0503 | 4,31E-12 | hypermethylated |
| NUP205 | 0,0502 | 1,15E-16 | hypermethylated |
| CCNDBP1 | 0,0497 | 1,61E-13 | hypermethylated |
| C1RL-AS1 | 0,0496 | 2,68E-13 | hypermethylated |
| NFKBIA | 0,0495 | 4,80E-10 | hypermethylated |
| DDX59 | 0,0495 | 7,27E-17 | hypermethylated |
| ILF2 | 0,0490 | 9,82E-11 | hypermethylated |
| SMYD4 | 0,0490 | 1,82E-10 | hypermethylated |
| SH3BP5 | 0,0486 | 1,95E-08 | hypermethylated |
| TMSB10 | 0,0479 | 4,87E-20 | hypermethylated |
| ACTR10 | 0,0479 | 4,36E-25 | hypermethylated |
| SNHG5 | 0,0476 | 1,12E-09 | hypermethylated |
| LINC00493 | 0,0476 | 6,82E-19 | hypermethylated |
| TOR1AIP2 | 0,0475 | 4,73E-18 | hypermethylated |
| ZNF833P | 0,0473 | 1,51E-08 | hypermethylated |
| NELFCD | 0,0473 | 7,76E-17 | hypermethylated |
| FAM187A | 0,0472 | 6,38E-09 | hypermethylated |
| MRPS14 | 0,0470 | 1,58E-14 | hypermethylated |
| ETV2 | 0,0468 | 9,38E-16 | hypermethylated |
| CGB7 | 0,0468 | 8,90E-13 | hypermethylated |
| DDX39A | 0,0467 | 2,26E-12 | hypermethylated |
| PCBP1 | 0,0465 | 4,03E-24 | hypermethylated |
| ORC1 | 0,0463 | 3,60E-25 | hypermethylated |
| DCTPP1 | 0,0457 | 2,06E-23 | hypermethylated |
| ZNF791 | 0,0456 | 7,34E-15 | hypermethylated |
| LPXN | 0,0456 | 2,30E-15 | hypermethylated |
| BCL2 | 0,0455 | 9,40E-15 | hypermethylated |
| SEC14L2 | 0,0455 | 9,94E-18 | hypermethylated |
| PIM1 | 0,0455 | 4,69E-10 | hypermethylated |
| HCG14 | 0,0455 | 4,25E-14 | hypermethylated |
| OBFC1 | 0,0454 | 8,73E-17 | hypermethylated |
| TAPSAR1 | 0,0452 | 2,17E-14 | hypermethylated |
| ZNF281 | 0,0451 | 2,02E-17 | hypermethylated |
| PPM1K | 0,0451 | 3,16E-09 | hypermethylated |
| ZNF720 | 0,0449 | 2,90E-15 | hypermethylated |
| TRAPPC6B | 0,0448 | 6,89E-14 | hypermethylated |
| ZNF497 | 0,0448 | 2,30E-11 | hypermethylated |
| ARF4 | 0,0446 | 9,59E-17 | hypermethylated |
| MIR132 | 0,0445 | 2,78E-13 | hypermethylated |
| HIC1 | 0,0444 | 9,64E-11 | hypermethylated |
| HYI | 0,0442 | 9,05E-15 | hypermethylated |
| ZFP36 | 0,0441 | 4,41E-15 | hypermethylated |
| CCBL1 | 0,0438 | 9,50E-20 | hypermethylated |
| MIR3610 | 0,0438 | 4,15E-20 | hypermethylated |
| RNU12 | 0,0437 | 1,81E-16 | hypermethylated |
| PAXIP1-AS2 | 0,0437 | 3,77E-15 | hypermethylated |
| NIFK-AS1 | -0,0439 | 1,91E-15 | hypomethylated |
| TINCR | -0,0441 | 2,20E-08 | hypomethylated |
| ZNF507 | -0,0449 | 1,15E-16 | hypomethylated |
| MAT2A | -0,0450 | 1,17E-15 | hypomethylated |
| QKI | -0,0454 | 2,75E-12 | hypomethylated |
| IFIH1 | -0,0458 | 3,74E-12 | hypomethylated |
| TRMT10B | -0,0460 | 3,04E-15 | hypomethylated |
| PPP1R21 | -0,0465 | 1,38E-17 | hypomethylated |
| DSEL | -0,0472 | 6,62E-09 | hypomethylated |
| NDUFS1 | -0,0480 | 1,22E-21 | hypomethylated |
| PSMC1 | -0,0486 | 1,41E-12 | hypomethylated |
| TRUB2 | -0,0487 | 3,75E-16 | hypomethylated |
| REEP3 | -0,0498 | 7,82E-17 | hypomethylated |
| MBTD1 | -0,0519 | 2,52E-12 | hypomethylated |
| MRPL42 | -0,0532 | 3,26E-15 | hypomethylated |
| NCOA5 | -0,0552 | 2,21E-15 | hypomethylated |
| MRPS11 | -0,0567 | 9,02E-15 | hypomethylated |
| SLC28A3 | -0,0576 | 4,41E-09 | hypomethylated |
| CCDC97 | -0,0596 | 1,83E-22 | hypomethylated |
| HSBP1L1 | -0,0600 | 1,02E-09 | hypomethylated |
| PPAPDC2 | -0,0601 | 1,08E-09 | hypomethylated |
| CRB3 | -0,0626 | 2,81E-09 | hypomethylated |
| WBSCR27 | -0,0640 | 1,09E-09 | hypomethylated |
| UGDH-AS1 | -0,0649 | 6,76E-09 | hypomethylated |
| MARVELD2 | -0,0656 | 4,45E-12 | hypomethylated |
| GPATCH1 | -0,0671 | 9,34E-22 | hypomethylated |
| BCL9L | -0,0704 | 7,68E-09 | hypomethylated |
| ZMYND8 | -0,0708 | 1,44E-08 | hypomethylated |
| C15orf61 | -0,0741 | 8,37E-10 | hypomethylated |
| MIR210HG | -0,0760 | 5,19E-13 | hypomethylated |
| ZNF563 | -0,0779 | 1,60E-16 | hypomethylated |
| SOWAHC | -0,0794 | 7,08E-09 | hypomethylated |
| SUSD2 | -0,0820 | 8,21E-10 | hypomethylated |
| ESRP2 | -0,0850 | 8,65E-10 | hypomethylated |
| SLC44A2 | -0,0870 | 7,60E-15 | hypomethylated |
| DRD3 | -0,0873 | 2,91E-09 | hypomethylated |
| PWWP2B | -0,0882 | 8,57E-12 | hypomethylated |
| GPD1 | -0,0918 | 2,45E-08 | hypomethylated |
| DNMBP-AS1 | -0,0968 | 1,50E-08 | hypomethylated |
| MIR210 | -0,0987 | 7,70E-19 | hypomethylated |
| KRT18 | -0,0999 | 3,74E-09 | hypomethylated |
| CLDN4 | -0,1035 | 9,60E-10 | hypomethylated |
| DNMBP | -0,1055 | 5,84E-10 | hypomethylated |
| SLC9A3R1 | -0,1061 | 8,03E-12 | hypomethylated |
| FAM86B3P | -0,1082 | 5,81E-10 | hypomethylated |
| MORN3 | -0,1085 | 6,73E-09 | hypomethylated |
| FAM53B-AS1 | -0,1087 | 2,46E-11 | hypomethylated |
| CAPS | -0,1094 | 4,33E-09 | hypomethylated |
| CATSPER2 | -0,1101 | 8,60E-19 | hypomethylated |
| NRN1L | -0,1159 | 1,51E-10 | hypomethylated |
| SKP1 | -0,1169 | 1,25E-08 | hypomethylated |
| POTEH | -0,1177 | 2,20E-08 | hypomethylated |
| ZSWIM5P2 | -0,1179 | 6,16E-09 | hypomethylated |
| TMEM191C | -0,1184 | 4,42E-09 | hypomethylated |
| BCAS1 | -0,1192 | 9,13E-09 | hypomethylated |
| LRRN3 | -0,1205 | 2,21E-08 | hypomethylated |
| CFI | -0,1259 | 1,39E-08 | hypomethylated |
| EIF2AK4 | -0,1317 | 4,24E-11 | hypomethylated |
| EDN2 | -0,1388 | 9,29E-09 | hypomethylated |
| MIR193A | -0,1391 | 2,10E-10 | hypomethylated |
| MUC20 | -0,1399 | 7,39E-14 | hypomethylated |
| SMCO3 | -0,1407 | 1,92E-12 | hypomethylated |
| GOLGA2P6 | -0,1446 | 1,02E-14 | hypomethylated |
| KCNRG | -0,1460 | 6,75E-09 | hypomethylated |
| AGAP2-AS1 | -0,1477 | 8,02E-09 | hypomethylated |
| CYP4F2 | -0,1488 | 2,35E-08 | hypomethylated |
| RNU6-1137P | -0,1500 | 6,57E-10 | hypomethylated |
| AKR1C4 | -0,1543 | 9,75E-11 | hypomethylated |
| ALG9-IT1 | -0,1548 | 1,29E-12 | hypomethylated |
| RN7SL67P | -0,1564 | 1,15E-08 | hypomethylated |
| LATS2-AS1 | -0,1574 | 5,94E-12 | hypomethylated |
| MIR517B | -0,1588 | 1,02E-09 | hypomethylated |
| ATP6V1G1P2 | -0,1596 | 1,56E-08 | hypomethylated |
| SIGLEC11 | -0,1602 | 1,06E-09 | hypomethylated |
| SMIM22 | -0,1602 | 2,82E-09 | hypomethylated |
| GRAPL | -0,1603 | 2,25E-10 | hypomethylated |
| VDAC1P11 | -0,1628 | 1,82E-19 | hypomethylated |
| TMPRSS11D | -0,1635 | 4,58E-09 | hypomethylated |
| MIR518B | -0,1679 | 4,11E-10 | hypomethylated |
| GPR37L1 | -0,1689 | 1,41E-14 | hypomethylated |
| POTED | -0,1713 | 6,39E-10 | hypomethylated |
| MIR33B | -0,1770 | 2,06E-12 | hypomethylated |
| LINC00226 | -0,1779 | 4,74E-09 | hypomethylated |
| LINC01085 | -0,1791 | 3,99E-11 | hypomethylated |
| PGAM1P5 | -0,1796 | 5,13E-10 | hypomethylated |
| MRS2P2 | -0,1799 | 1,31E-30 | hypomethylated |
| CENPB | -0,1821 | 3,53E-09 | hypomethylated |
| KRTAP2-4 | -0,1836 | 4,14E-09 | hypomethylated |
| LINC00028 | -0,1840 | 1,25E-09 | hypomethylated |
| SULT1C3 | -0,1857 | 4,21E-09 | hypomethylated |
| C1orf64 | -0,1866 | 3,92E-11 | hypomethylated |
| CEACAM20 | -0,1878 | 1,22E-09 | hypomethylated |
| MIR3180-4 | -0,1895 | 2,04E-14 | hypomethylated |
| HCCAT3 | -0,1979 | 2,30E-10 | hypomethylated |
| ALG1L13P | -0,1995 | 3,00E-12 | hypomethylated |
| KARSP2 | -0,2095 | 4,84E-14 | hypomethylated |
| SPTY2D1-AS1 | -0,2135 | 2,86E-12 | hypomethylated |
| MYH2 | -0,2145 | 5,26E-10 | hypomethylated |
| C1orf168 | -0,2146 | 3,25E-09 | hypomethylated |
| RN7SL358P | -0,2156 | 1,69E-09 | hypomethylated |
| FOXN3-AS2 | -0,2181 | 4,13E-12 | hypomethylated |
| OR52A1 | -0,2186 | 9,27E-09 | hypomethylated |
| PSG1 | -0,2208 | 1,09E-10 | hypomethylated |
| MIR568 | -0,2249 | 3,98E-11 | hypomethylated |
| NLRP9 | -0,2289 | 2,38E-08 | hypomethylated |
| RNU6-45P | -0,2296 | 2,64E-17 | hypomethylated |
| ATP5HP1 | -0,2307 | 2,54E-19 | hypomethylated |
| MIR320D1 | -0,2321 | 4,73E-17 | hypomethylated |
| CCDC168 | -0,2394 | 1,64E-08 | hypomethylated |
| KLKP1 | -0,2508 | 5,99E-09 | hypomethylated |
| OR51B2 | -0,2566 | 1,81E-08 | hypomethylated |
| SMN1 | -0,2685 | 9,05E-26 | hypomethylated |
| SNORA8 | -0,2704 | 3,45E-15 | hypomethylated |
| MYO16-AS1 | -0,2777 | 3,18E-10 | hypomethylated |
| LINC01071 | -0,2780 | 2,13E-12 | hypomethylated |
| TMPRSS11BNL | -0,2780 | 3,52E-14 | hypomethylated |
| KRTAP15-1 | -0,2790 | 1,36E-09 | hypomethylated |
| RGPD2 | -0,2938 | 3,93E-22 | hypomethylated |
| JRKL-AS1 | -0,2997 | 7,35E-09 | hypomethylated |
| SCARNA6 | -0,3082 | 9,78E-11 | hypomethylated |
| MIR548B | -0,3431 | 1,30E-15 | hypomethylated |
| TPTE2P3 | -0,3525 | 1,83E-18 | hypomethylated |
| C1orf189 | -0,4121 | 6,92E-35 | hypomethylated |
| HS1BP3-IT1 | -0,4148 | 3,88E-38 | hypomethylated |

**Supplementary Table 4 List of the 250 top-ranking differentially methylated genes between Stage I BrCa and normal tissues.**

| **Symbol** | **Mean Methylation difference** | **FDR** | **Methylation status in BrCa stage I in relation to normal** |
| --- | --- | --- | --- |
| CHL1-AS2 | 0,1512 | 3,75E-24 | hypermethylated |
| GRM1 | 0,1517 | 2,01E-27 | hypermethylated |
| FAT4 | 0,1517 | 1,17E-29 | hypermethylated |
| ST8SIA3 | 0,1520 | 2,32E-33 | hypermethylated |
| STMN2 | 0,1523 | 8,30E-28 | hypermethylated |
| SALL2 | 0,1533 | 8,42E-27 | hypermethylated |
| SNCA | 0,1535 | 4,07E-27 | hypermethylated |
| ZDBF2 | 0,1540 | 6,80E-25 | hypermethylated |
| PHYHIPL | 0,1542 | 1,10E-36 | hypermethylated |
| ITPRIPL1 | 0,1543 | 4,80E-31 | hypermethylated |
| IGLON5 | 0,1545 | 2,31E-28 | hypermethylated |
| BHMT2 | 0,1551 | 9,37E-27 | hypermethylated |
| C14orf23 | 0,1559 | 1,66E-24 | hypermethylated |
| GSC2 | 0,1561 | 3,41E-26 | hypermethylated |
| MIR124-3 | 0,1562 | 7,65E-29 | hypermethylated |
| FOXB2 | 0,1562 | 4,19E-25 | hypermethylated |
| AKR1B1 | 0,1563 | 2,50E-28 | hypermethylated |
| ZNF334 | 0,1565 | 1,68E-37 | hypermethylated |
| CR1 | 0,1566 | 3,47E-31 | hypermethylated |
| NPY | 0,1570 | 2,88E-34 | hypermethylated |
| EDNRB | 0,1582 | 1,12E-32 | hypermethylated |
| HNF1B | 0,1584 | 1,19E-25 | hypermethylated |
| ZNF667-AS1 | 0,1584 | 6,99E-28 | hypermethylated |
| MIR9-3 | 0,1592 | 6,33E-26 | hypermethylated |
| MGARP | 0,1594 | 3,29E-28 | hypermethylated |
| FAM19A3 | 0,1600 | 6,39E-33 | hypermethylated |
| C8orf88 | 0,1607 | 1,11E-39 | hypermethylated |
| ZNF578 | 0,1611 | 5,58E-26 | hypermethylated |
| SYNDIG1 | 0,1615 | 1,81E-28 | hypermethylated |
| KCNE3 | 0,1637 | 3,56E-28 | hypermethylated |
| NKX2-1-AS1 | 0,1641 | 7,29E-26 | hypermethylated |
| SLC35G2 | 0,1643 | 3,72E-24 | hypermethylated |
| CA3 | 0,1643 | 8,53E-29 | hypermethylated |
| EPHA5 | 0,1643 | 6,71E-28 | hypermethylated |
| DGAT2L7P | 0,1666 | 1,31E-23 | hypermethylated |
| SENCR | 0,1672 | 7,23E-27 | hypermethylated |
| CD34 | 0,1673 | 5,04E-30 | hypermethylated |
| DLG5-AS1 | 0,1679 | 2,59E-27 | hypermethylated |
| CYP2T2P | 0,1696 | 1,35E-30 | hypermethylated |
| ATOH1 | 0,1722 | 6,71E-26 | hypermethylated |
| C6orf58 | 0,1728 | 1,61E-25 | hypermethylated |
| TTYH1 | 0,1735 | 4,07E-27 | hypermethylated |
| GPR149 | 0,1743 | 1,16E-29 | hypermethylated |
| HTR1A | 0,1743 | 2,93E-28 | hypermethylated |
| UGT3A2 | 0,1747 | 1,15E-32 | hypermethylated |
| GNG11 | 0,1758 | 3,00E-23 | hypermethylated |
| LHFPL4 | 0,1759 | 5,92E-37 | hypermethylated |
| UBA7 | 0,1766 | 1,70E-27 | hypermethylated |
| CRCT1 | 0,1773 | 1,60E-27 | hypermethylated |
| PCSK1 | 0,1775 | 8,00E-30 | hypermethylated |
| ZNF559-ZNF177 | 0,1777 | 9,51E-36 | hypermethylated |
| LINC00202-1 | 0,1778 | 7,27E-26 | hypermethylated |
| GYPC | 0,1780 | 2,76E-24 | hypermethylated |
| OLIG2 | 0,1784 | 1,90E-24 | hypermethylated |
| MOS | 0,1784 | 5,69E-30 | hypermethylated |
| OLIG3 | 0,1791 | 3,38E-26 | hypermethylated |
| PRLHR | 0,1793 | 5,32E-36 | hypermethylated |
| GABRA4 | 0,1797 | 1,13E-39 | hypermethylated |
| UNCX | 0,1799 | 1,62E-31 | hypermethylated |
| VSX1 | 0,1801 | 6,26E-30 | hypermethylated |
| OTX2 | 0,1803 | 1,64E-30 | hypermethylated |
| ADAMTS20 | 0,1804 | 9,40E-30 | hypermethylated |
| CYP2A13 | 0,1830 | 4,97E-31 | hypermethylated |
| MSC | 0,1836 | 5,57E-25 | hypermethylated |
| QRFPR | 0,1844 | 8,46E-26 | hypermethylated |
| OTP | 0,1847 | 1,04E-26 | hypermethylated |
| ZFHX4-AS1 | 0,1847 | 1,18E-25 | hypermethylated |
| POU3F3 | 0,1865 | 2,47E-24 | hypermethylated |
| CHST2 | 0,1866 | 1,27E-30 | hypermethylated |
| LHX9 | 0,1866 | 7,37E-24 | hypermethylated |
| RBM27 | 0,1866 | 6,67E-31 | hypermethylated |
| PTF1A | 0,1868 | 5,90E-31 | hypermethylated |
| HIST2H3D | 0,1871 | 1,61E-30 | hypermethylated |
| NEUROG3 | 0,1884 | 8,68E-31 | hypermethylated |
| ONECUT2 | 0,1886 | 7,27E-30 | hypermethylated |
| CCDC140 | 0,1896 | 5,08E-25 | hypermethylated |
| RECK | 0,1902 | 4,20E-24 | hypermethylated |
| CHRND | 0,1904 | 1,46E-26 | hypermethylated |
| VAX1 | 0,1904 | 1,51E-23 | hypermethylated |
| INA | 0,1904 | 1,25E-36 | hypermethylated |
| WT1-AS | 0,1910 | 3,13E-24 | hypermethylated |
| PRDM13 | 0,1913 | 2,66E-33 | hypermethylated |
| NPBWR1 | 0,1917 | 1,50E-29 | hypermethylated |
| KCTD8 | 0,1918 | 1,07E-30 | hypermethylated |
| ZIC4 | 0,1924 | 4,62E-27 | hypermethylated |
| GRASP | 0,1931 | 4,41E-37 | hypermethylated |
| FAM90A28P | 0,1936 | 6,17E-25 | hypermethylated |
| USP44 | 0,1938 | 9,67E-31 | hypermethylated |
| TLX1 | 0,1938 | 2,65E-23 | hypermethylated |
| GALR3 | 0,1938 | 2,54E-24 | hypermethylated |
| ZNF571-AS1 | 0,1943 | 1,40E-40 | hypermethylated |
| FEZF2 | 0,1947 | 1,16E-28 | hypermethylated |
| LHX8 | 0,1949 | 3,47E-25 | hypermethylated |
| FZD7 | 0,1960 | 5,24E-25 | hypermethylated |
| TLX2 | 0,1961 | 4,54E-26 | hypermethylated |
| PDX1 | 0,1961 | 4,72E-31 | hypermethylated |
| L1TD1 | 0,1963 | 6,14E-28 | hypermethylated |
| SFTA3 | 0,1969 | 2,96E-31 | hypermethylated |
| GHSR | 0,1971 | 2,11E-44 | hypermethylated |
| ZNF560 | 0,1971 | 3,49E-39 | hypermethylated |
| TBX15 | 0,1984 | 7,76E-29 | hypermethylated |
| TFAP2D | 0,1986 | 9,94E-29 | hypermethylated |
| NKX2-1 | 0,1986 | 2,11E-29 | hypermethylated |
| FSIP2 | 0,1990 | 4,57E-29 | hypermethylated |
| ZIC1 | 0,1991 | 3,64E-26 | hypermethylated |
| MIR663A | 0,1992 | 3,09E-39 | hypermethylated |
| CDX2 | 0,1995 | 2,18E-31 | hypermethylated |
| HOXD11 | 0,1997 | 2,30E-26 | hypermethylated |
| FAM162B | 0,1998 | 1,34E-24 | hypermethylated |
| ACTL6B | 0,1999 | 1,21E-40 | hypermethylated |
| TLX3 | 0,2002 | 3,19E-26 | hypermethylated |
| ZNF132 | 0,2004 | 1,79E-24 | hypermethylated |
| HOXD-AS2 | 0,2005 | 6,69E-28 | hypermethylated |
| SLITRK1 | 0,2006 | 6,53E-28 | hypermethylated |
| SOX14 | 0,2007 | 1,53E-36 | hypermethylated |
| ZNF876P | 0,2013 | 7,68E-27 | hypermethylated |
| ZNF177 | 0,2014 | 1,94E-40 | hypermethylated |
| ACTA1 | 0,2014 | 6,77E-33 | hypermethylated |
| EVX2 | 0,2022 | 9,39E-27 | hypermethylated |
| DNASE1L2 | 0,2026 | 4,86E-29 | hypermethylated |
| SERPING1 | 0,2031 | 6,56E-35 | hypermethylated |
| AVPR1A | 0,2031 | 5,31E-35 | hypermethylated |
| DMRTA2 | 0,2035 | 1,01E-29 | hypermethylated |
| MSX2P1 | 0,2047 | 2,19E-33 | hypermethylated |
| HSPB6 | 0,2047 | 5,05E-25 | hypermethylated |
| ENPP2 | 0,2057 | 1,41E-32 | hypermethylated |
| YWHAEP7 | 0,2061 | 2,09E-26 | hypermethylated |
| HOXA4 | 0,2074 | 2,90E-27 | hypermethylated |
| LHX1 | 0,2076 | 2,29E-27 | hypermethylated |
| NXPH1 | 0,2078 | 7,40E-33 | hypermethylated |
| C12orf68 | 0,2083 | 2,85E-38 | hypermethylated |
| FOXA2 | 0,2085 | 7,50E-31 | hypermethylated |
| UCN | 0,2095 | 2,30E-29 | hypermethylated |
| HMX2 | 0,2096 | 1,19E-30 | hypermethylated |
| CARTPT | 0,2102 | 2,11E-29 | hypermethylated |
| PHOX2B | 0,2115 | 1,08E-27 | hypermethylated |
| SSTR1 | 0,2118 | 5,59E-32 | hypermethylated |
| PRSS30P | 0,2122 | 1,92E-35 | hypermethylated |
| WT1 | 0,2130 | 5,41E-31 | hypermethylated |
| NETO1 | 0,2133 | 1,25E-41 | hypermethylated |
| RESP18 | 0,2134 | 7,86E-38 | hypermethylated |
| CFTR | 0,2140 | 1,35E-26 | hypermethylated |
| SOX1 | 0,2145 | 5,03E-34 | hypermethylated |
| SLC32A1 | 0,2153 | 1,18E-35 | hypermethylated |
| LINC00966 | 0,2161 | 3,34E-28 | hypermethylated |
| YBX3P1 | 0,2171 | 1,94E-35 | hypermethylated |
| NKX2-6 | 0,2176 | 1,32E-35 | hypermethylated |
| CCDC36 | 0,2182 | 2,61E-32 | hypermethylated |
| C14orf39 | 0,2186 | 1,13E-31 | hypermethylated |
| MAGI2-AS3 | 0,2191 | 2,41E-38 | hypermethylated |
| CDO1 | 0,2195 | 2,07E-37 | hypermethylated |
| VSTM2B | 0,2196 | 1,93E-37 | hypermethylated |
| HTR1B | 0,2214 | 2,49E-39 | hypermethylated |
| ANGPTL2 | 0,2215 | 4,37E-36 | hypermethylated |
| GALR1 | 0,2227 | 3,81E-30 | hypermethylated |
| CXCL1P | 0,2229 | 9,32E-27 | hypermethylated |
| NES | 0,2252 | 9,54E-31 | hypermethylated |
| PCDH8 | 0,2255 | 3,82E-31 | hypermethylated |
| NRIP2 | 0,2267 | 6,51E-25 | hypermethylated |
| FOXD4 | 0,2300 | 1,64E-34 | hypermethylated |
| FOXQ1 | 0,2313 | 3,16E-35 | hypermethylated |
| ALX1 | 0,2315 | 1,55E-35 | hypermethylated |
| SCGB1B2P | 0,2319 | 2,46E-33 | hypermethylated |
| BARHL2 | 0,2321 | 4,85E-35 | hypermethylated |
| TBX18 | 0,2324 | 1,37E-36 | hypermethylated |
| HIST3H2BA | 0,2326 | 1,77E-29 | hypermethylated |
| NMBR | 0,2339 | 2,42E-38 | hypermethylated |
| DRD5 | 0,2346 | 5,41E-31 | hypermethylated |
| SST | 0,2346 | 9,52E-43 | hypermethylated |
| NKX2-2 | 0,2351 | 6,19E-30 | hypermethylated |
| POU4F3 | 0,2361 | 4,29E-39 | hypermethylated |
| ASCL4 | 0,2365 | 1,09E-24 | hypermethylated |
| NEFM | 0,2372 | 4,76E-49 | hypermethylated |
| FCAR | 0,2379 | 1,33E-34 | hypermethylated |
| SLC18A3 | 0,2398 | 1,84E-44 | hypermethylated |
| HOXD12 | 0,2414 | 2,51E-39 | hypermethylated |
| FOXE3 | 0,2436 | 5,18E-36 | hypermethylated |
| TCF24 | 0,2440 | 1,86E-38 | hypermethylated |
| SKOR2 | 0,2441 | 4,96E-47 | hypermethylated |
| LINC01077 | 0,2441 | 2,19E-25 | hypermethylated |
| MIR9-1 | 0,2450 | 1,49E-30 | hypermethylated |
| ABCB10P4 | 0,2470 | 4,26E-46 | hypermethylated |
| IFNL4P1 | 0,2478 | 1,18E-35 | hypermethylated |
| C9orf172 | 0,2479 | 9,25E-38 | hypermethylated |
| NEFH | 0,2485 | 8,29E-43 | hypermethylated |
| SIX6 | 0,2487 | 9,35E-35 | hypermethylated |
| DSC3 | 0,2495 | 4,34E-36 | hypermethylated |
| NKX2-4 | 0,2511 | 1,08E-40 | hypermethylated |
| GLIPR1L1 | 0,2512 | 6,85E-46 | hypermethylated |
| UNC93B7 | 0,2513 | 1,77E-41 | hypermethylated |
| ZNF154 | 0,2518 | 1,42E-25 | hypermethylated |
| RHOQP2 | 0,2519 | 4,39E-43 | hypermethylated |
| DBX1 | 0,2524 | 1,17E-39 | hypermethylated |
| CPXM1 | 0,2560 | 3,46E-39 | hypermethylated |
| SPARC | 0,2564 | 5,41E-32 | hypermethylated |
| NKX1-1 | 0,2581 | 2,32E-40 | hypermethylated |
| HOXD8 | 0,2584 | 6,71E-26 | hypermethylated |
| OXT | 0,2586 | 1,80E-27 | hypermethylated |
| ATXN8OS | 0,2598 | 5,57E-40 | hypermethylated |
| NKX6-2 | 0,2599 | 3,18E-43 | hypermethylated |
| POU4F1 | 0,2606 | 3,46E-35 | hypermethylated |
| UTF1 | 0,2619 | 1,99E-33 | hypermethylated |
| NKAPL | 0,2621 | 5,61E-42 | hypermethylated |
| FOXG1 | 0,2625 | 1,55E-35 | hypermethylated |
| SOX17 | 0,2627 | 4,25E-41 | hypermethylated |
| SYNE1-AS1 | 0,2629 | 6,92E-32 | hypermethylated |
| GABRG2 | 0,2639 | 8,32E-40 | hypermethylated |
| RNU5F-2P | 0,2653 | 1,56E-24 | hypermethylated |
| HOTAIRM1 | 0,2662 | 3,34E-41 | hypermethylated |
| PRAP1 | 0,2663 | 1,24E-36 | hypermethylated |
| MED15P3 | 0,2668 | 7,21E-35 | hypermethylated |
| BHLHE23 | 0,2684 | 1,67E-40 | hypermethylated |
| ELTD1 | 0,2719 | 1,81E-41 | hypermethylated |
| GSX1 | 0,2774 | 1,35E-40 | hypermethylated |
| CLEC14A | 0,2808 | 1,54E-39 | hypermethylated |
| FOXD3 | 0,2814 | 9,39E-31 | hypermethylated |
| HIST1H4F | 0,2824 | 3,63E-30 | hypermethylated |
| ZNF728 | 0,2825 | 1,70E-43 | hypermethylated |
| HIST2H3PS2 | 0,2872 | 7,65E-51 | hypermethylated |
| NEUROD1 | 0,2874 | 1,31E-47 | hypermethylated |
| CCDC181 | 0,2881 | 6,67E-47 | hypermethylated |
| KCNJ2-AS1 | 0,2891 | 1,64E-42 | hypermethylated |
| POU4F2 | 0,2905 | 7,02E-52 | hypermethylated |
| PDIA3P1 | 0,2910 | 1,27E-33 | hypermethylated |
| OR2I1P | 0,2912 | 5,42E-35 | hypermethylated |
| HIST1H1A | 0,2941 | 4,63E-28 | hypermethylated |
| COX11P1 | 0,2980 | 8,05E-51 | hypermethylated |
| NKX2-2-AS1 | 0,3003 | 9,65E-35 | hypermethylated |
| TAC1 | 0,3041 | 1,12E-40 | hypermethylated |
| DEFB132 | 0,3048 | 8,97E-28 | hypermethylated |
| PRAC1 | 0,3109 | 2,57E-31 | hypermethylated |
| TRH | 0,3177 | 3,97E-42 | hypermethylated |
| MIR129-2 | 0,3198 | 1,71E-42 | hypermethylated |
| FERD3L | 0,3404 | 5,17E-54 | hypermethylated |
| MIR124-2 | 0,3551 | 1,18E-35 | hypermethylated |
| IGJ | 0,3753 | 6,85E-39 | hypermethylated |
| COX7A1 | 0,3755 | 3,21E-48 | hypermethylated |
| SNORD52 | -0,1657 | 1,32E-32 | hypomethylated |
| LGALS9B | -0,1788 | 1,03E-28 | hypomethylated |
| TSTD1 | -0,2132 | 1,55E-43 | hypomethylated |
| SLAMF7 | -0,2165 | 4,43E-29 | hypomethylated |
| BPIFA4P | -0,2471 | 3,94E-40 | hypomethylated |
| BRWD1-AS1 | -0,2499 | 3,09E-36 | hypomethylated |
| MIR21 | -0,2554 | 3,47E-45 | hypomethylated |
| CHRM3-AS2 | -0,2757 | 7,14E-36 | hypomethylated |
| REG1P | -0,3287 | 1,27E-35 | hypomethylated |
| DEFB118 | -0,3368 | 4,24E-47 | hypomethylated |
| OR2M3 | -0,3391 | 1,02E-41 | hypomethylated |
| FGF12-AS1 | -0,3471 | 6,10E-38 | hypomethylated |
| OR2M7 | -0,3634 | 3,88E-44 | hypomethylated |

**Supplementary Table 5 Genes counts and enrichment percent provided by DAVID tool between stage I BrCa and normal breast tissues**

| **Biological Process** | **Genes Count** | **% Enrichment** | **P-Value** | **Benjamini** |
| --- | --- | --- | --- | --- |
| negative regulation of transcription from RNA polymerase II promoter | 30 | 12,4 | 3,10E-10 | 3,40E-07 |
| transcription from RNA polymerase II promoter | 25 | 10,4 | 6,60E-10 | 3,60E-07 |
| regulation of transcription, DNA-templated | 42 | 17,4 | 6,10E-09 | 1,70E-06 |
| positive regulation of transcription from RNA polymerase II promoter | 33 | 13,7 | 6,30E-09 | 1,70E-06 |
| neuropeptide signaling pathway | 11 | 4,6 | 9,40E-08 | 2,00E-05 |
| transcription, DNA-templated | 46 | 19,1 | 1,50E-07 | 2,80E-05 |
| endocrine pancreas development | 7 | 2,9 | 2,20E-07 | 3,40E-05 |
| chemical synaptic transmission | 14 | 5,8 | 1,30E-06 | 1,80E-04 |
| regulation of transcription from RNA polymerase II promoter | 15 | 6,2 | 2,10E-04 | 2,40E-02 |
| positive regulation of renal sodium excretion | 4 | 1,7 | 2,20E-04 | 2,40E-02 |
| neuron fate specification | 4 | 1,7 | 3,70E-04 | 3,60E-02 |
| **Molecular Function** | **Genes Count** | **% Enrichment** | **P-Value** | **Benjamini** |
| sequence-specific DNA binding | 41 | 17 | 5,50E-24 | 1,30E-21 |
| transcription factor activity, sequence-specific DNA binding | 35 | 14,5 | 2,00E-10 | 2,40E-08 |
| transcriptional activator activity, RNA polymerase II core promoter proximal region sequence-specific binding | 16 | 6,6 | 2,30E-08 | 1,70E-06 |
| RNA polymerase II core promoter proximal region sequence-specific DNA binding | 19 | 7,9 | 2,90E-08 | 1,70E-06 |
| RNA polymerase II transcription factor activity, sequence-specific DNA binding | 11 | 4,6 | 1,10E-05 | 5,40E-04 |
| RNA polymerase II regulatory region sequence-specific DNA binding | 11 | 4,6 | 6,10E-05 | 2,40E-03 |
| peptide hormone binding | 5 | 2,1 | 1,60E-04 | 4,90E-03 |
| protein dimerization activity | 9 | 3,7 | 1,60E-04 | 4,90E-03 |
| transcription factor activity, RNA polymerase II distal enhancer sequence-specific binding | 6 | 2,5 | 5,90E-04 | 1,60E-02 |
| neuropeptide binding | 4 | 1,7 | 1,10E-03 | 2,60E-02 |
| DNA binding | 31 | 12,9 | 1,90E-03 | 4,10E-02 |
|  |  |  |  |  |

**Supplementary Table 6 Genes involved in the pathophysiology of Breast Cancer according to the literature.**

| **UniProt ID** | **Gene** | **Protein** | **Reference** |
| --- | --- | --- | --- |
| **P38398** | **BRCA1** | Breast cancer type 1 susceptibility protein | (Narod and Salmena 2011) |
| **A0A248X3Z0** | **BRCA2** | Breast cancer type 2 susceptibility protein | (Narod and Salmena 2011) |
| **Q9NS23** | **RASSF1** | Ras association domain-containing protein 1 | (Li, Wang et al. 2019) |
| **P03372** | **ESR1** | Estrogen Receptor 1 | (Dustin, Gu et al. 2019) |
| **P04637** | **TP53** | Cellular tumor antigen p53 | (Li, Chen et al. 2020) |
| **P42336** | **PIK3CA** | Phosphatidylinositol-4,5-Bisphosphate 3-Kinase Catalytic Subunit Alpha | (Arsenic, Lehmann et al. 2014) |
| **Q9HCU9** | **BRMS1** | Breast Cancer Metastasis Suppressor | (Zhang, Ye et al. 2014) |
| **P12830** | **CDH1** | Cadherin | (Corso, Veronesi et al. 2018) |
| **Q15828** | **CST6** | Cystatin E/M | (Chimonidou, Tzitzira et al. 2013) |
| **P60484** | **PTEN** | Phosphatidylinositol 3,4,5-trisphosphate 3-phosphatase and dual-specificity protein phosphatase | (Carbognin, Miglietta et al. 2019) |

Narod, S.A. and L. Salmena, BRCA1 and BRCA2 mutations and breast cancer. Discov Med, 2011. 12(66): p. 445-53.

Li, M., et al., Diagnostic value of RASSF1A methylation for breast cancer: a meta-analysis. Biosci Rep, 2019. 39(6).

Dustin, D., G. Gu, and S.A.W. Fuqua, ESR1 mutations in breast cancer. Cancer, 2019. 125(21): p. 3714-3728.

Li, X., et al., Impact of TP53 mutations in breast cancer: Clinicopathological features and prognosisImpact of TP53 mutations in breast CA. Thorac Cancer, 2020. 11(7): p. 1861-1868.

Arsenic, R., et al., Analysis of PIK3CA mutations in breast cancer subtypes. Appl Immunohistochem Mol Morphol, 2014. 22(1): p. 50-6.

Zhang, Y., et al., Expression of breast cancer metastasis suppressor-1, BRMS-1, in human breast cancer and the biological impact of BRMS-1 on the migration of breast cancer cells. Anticancer Res, 2014. 34(3): p. 1417-26.

Corso, G., et al., Prognosis and outcome in CDH1-mutant lobular breast cancer. Eur J Cancer Prev, 2018. 27(3): p. 237-238.

Carbognin, L., et al., Prognostic and Predictive Implications of PTEN in Breast Cancer: Unfulfilled Promises but Intriguing Perspectives. Cancers (Basel), 2019. 11(9).

Chimonidou, M., et al., CST6 promoter methylation in circulating cell-free DNA of breast cancer patients. Clinical Biochemistry, 2013. 46(3): p. 235-240.
