# Supplementary figures and images for: Deciphering the Methylation Landscape in Breast Cancer: Diagnostic and Prognostic Biosignatures through Automated Machine Learning
Source: Cancers (Basel). 2021 Apr 2;13(7):1677. doi: 10.3390/cancers13071677 (PMC8037759; doi:10.3390/cancers13071677)

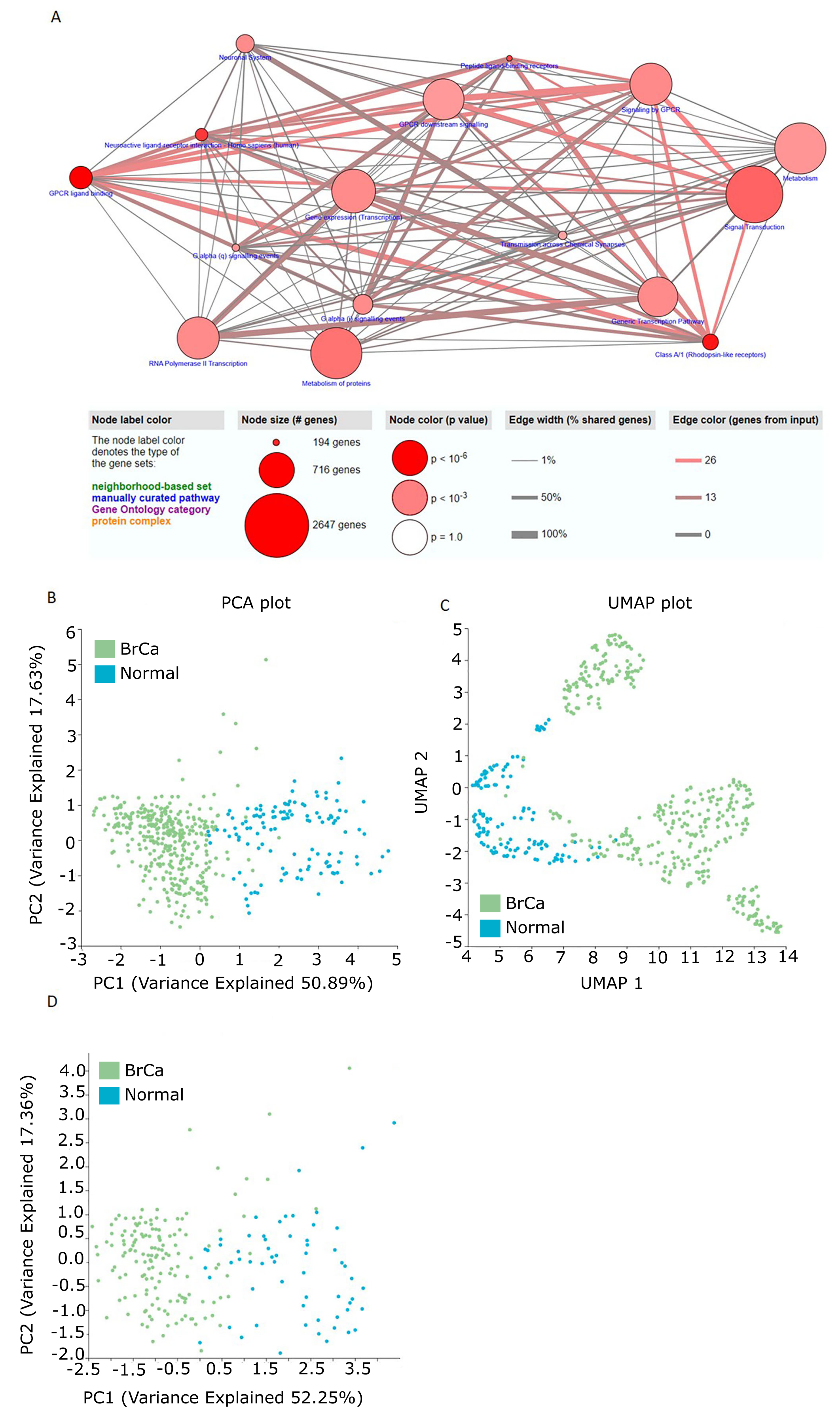

Supplement: Supplementary file 1 [file cancers-13-01677-s001.zip › Suppementary figures/S1.tif]

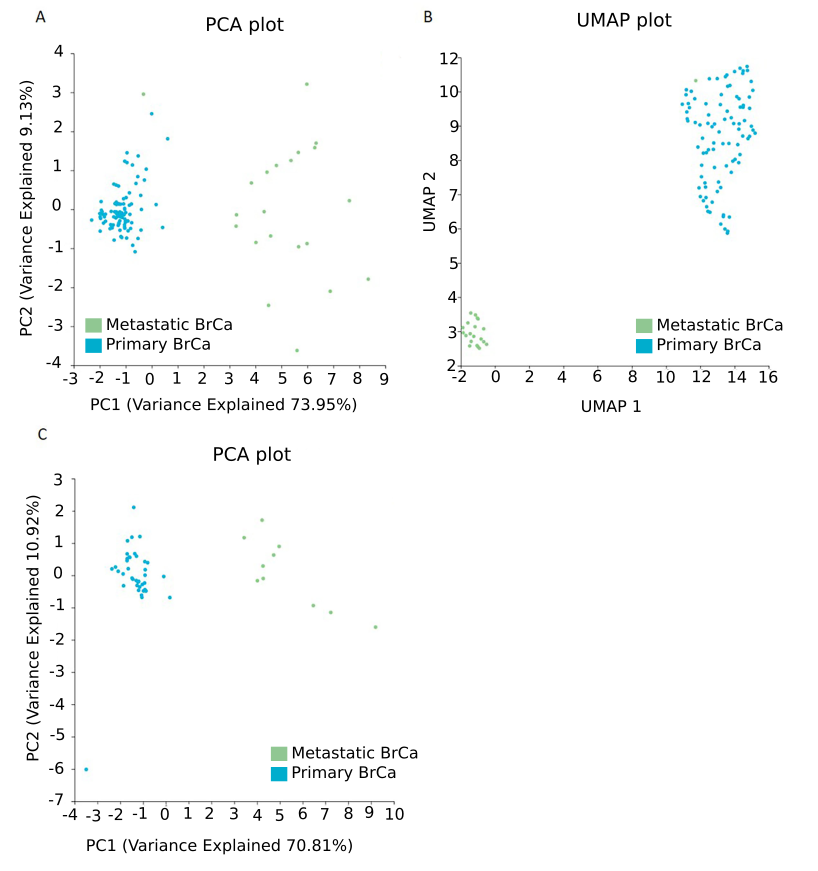

Supplement: Supplementary file 1 [file cancers-13-01677-s001.zip › Suppementary figures/S2.tif]

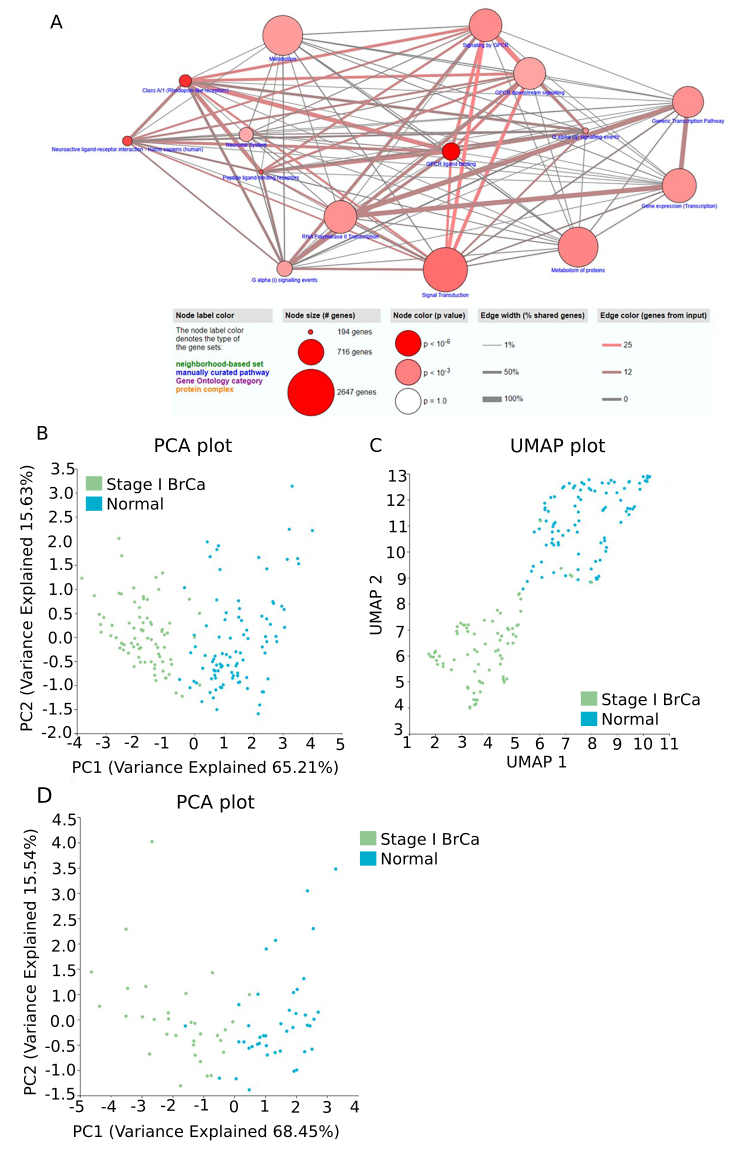

Supplement: Supplementary file 1 [file cancers-13-01677-s001.zip › Suppementary figures/S3.tif]

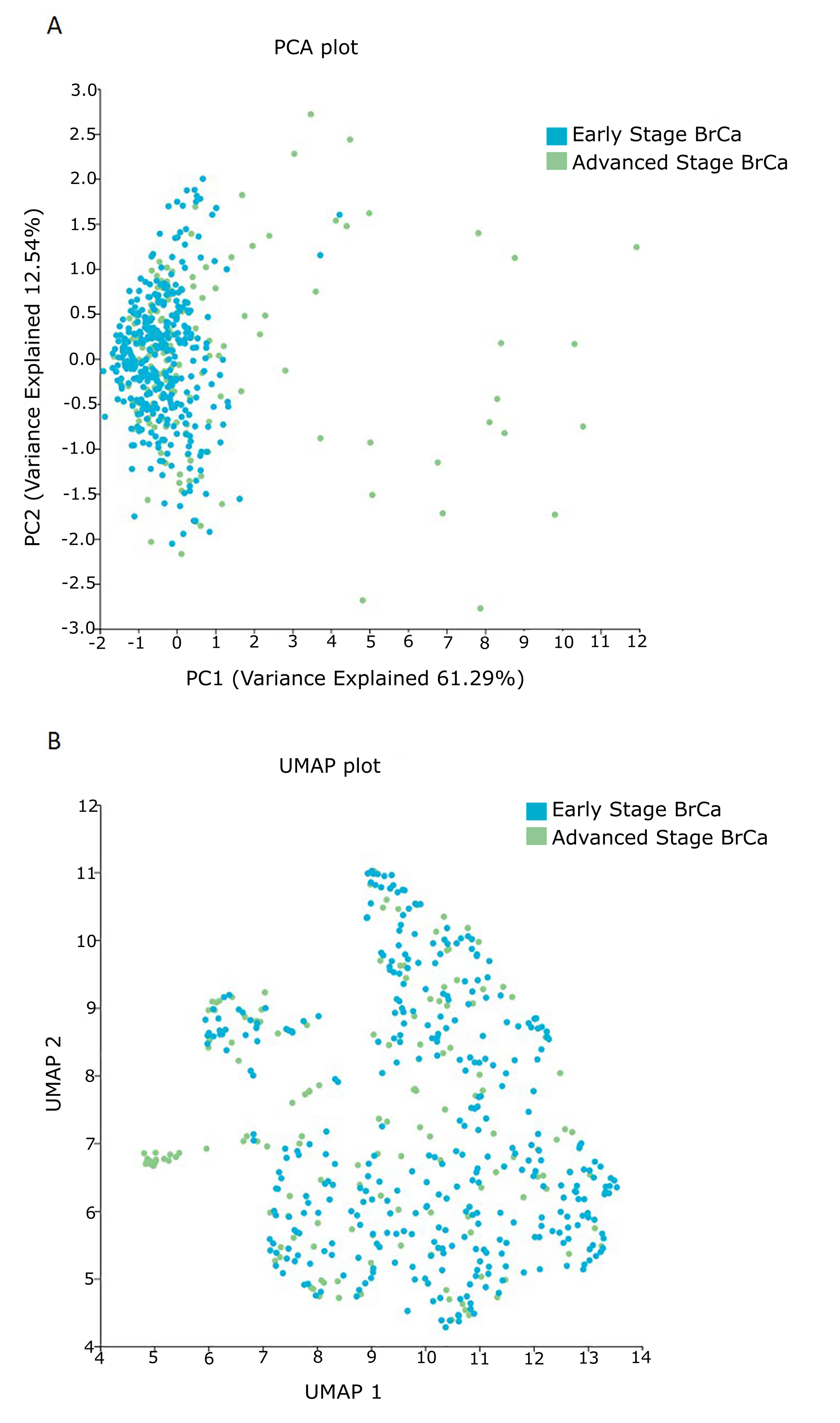

Supplement: Supplementary file 1 [file cancers-13-01677-s001.zip › Suppementary figures/S4.tif]
